# LncRNA THUMPD3‐AS1 Regulates Behavioral and Synaptic Structural Abnormalities in Schizophrenia via miR‐485‐5p and ARHGAP8

**DOI:** 10.1002/advs.202508867

**Published:** 2025-10-30

**Authors:** Xiaojuan Gong, Lingxi Chen, Xin Guo, Anna Jiang, Yayi He, Chunxia Yan, Liang Ma, Jiayang Gao, Jinyu Zhang, Bao Zhang

**Affiliations:** ^1^ College of Medicine and Forensic Xi'an Jiaotong University Health Science Center Xi'an Shaanxi 710061 China; ^2^ Bio‐evidence Sciences Academy Xi'an Jiaotong University Xi'an Shaanxi 710049 China; ^3^ Department of Computer Science City University of Hong Kong Kowloon Hong Kong 999077 China; ^4^ Department of Biomedical Sciences City University of Hong Kong Kowloon Hong Kong 999077 China; ^5^ OmicLab Limited Unit 917, 19 Science Park West Avenue, New Territories Hong Kong 999077 China; ^6^ School of Aerospace Engineering Xi'an Jiaotong University Xi'an Shaanxi 710049 China; ^7^ Department of Endocrinology The First Affiliated Hospital of Xi'an Jiaotong University Xi'an Shaanxi 710061 China; ^8^ Biggs Institute for Alzheimer's and Neurodegenerative Disease Department of Pharmacology The University of Texas Health Science Center at San Antonio San Antonio 78229 USA

**Keywords:** noncoding RNA regulation, Rho GTPase signaling, schizophrenia, synaptic pathology

## Abstract

Schizophrenia (SCZ) is characterized by synaptic structural deficits, yet how dysregulated noncoding RNAs (ncRNAs) drive these abnormalities remains unknown. Through integrative multilayered analysis of SCZ data from whole transcriptome sequencing (blood samples), GWAS risk loci, and expression data using pipeline ceRNAxis, the THUMPD3‐AS1/miR‐485‐5p/ARHGAP8 axis is identified as a key regulator of synaptic function. Functional validation reveals that THUMPD3‐AS1 acts as a competitive endogenous RNA, sequestering miR‐485‐5p and thereby derepressing ARHGAP8. Despite suppressing RhoA activity, ARHGAP8 enhances ROCK2 activation through RhoB/C‐mediated compensatory mechanisms. Hyperactivation of ROCK2 through this noncanonical pathway disrupted actin cytoskeletal remodeling patterns, leading to increased immature dendritic spines and synaptic ultrastructural defects, which are pathological features associated with SCZ. In vivo, ventral hippocampal (vHip) overexpression of miR‐485‐5p or targeted knockdown of THUMPD3‐AS1 rescued MK‐801‐induced SCZ‐like phenotypes (anxiety, cognitive deficits, and social memory impairments) and restored synaptic ultrastructure. Crucially, this regulatory axis is cross‐species conservation, with bidirectional expression changes validated in patient‐derived blood and vHip tissues of mice. The findings reveal a novel ncRNA‐driven pathogenic cascade in SCZ, where dysregulated RhoB/C‐ROCK2 signaling, distinct from classical RhoA pathways, mediates synaptic destabilization. This presents a therapeutic axis for precision interventions targeting noncanonical actin cytoskeletal remodeling.

## Graphical abstract

Schizophrenia (SCZ) is linked to synaptic structural deficits driven by dysregulated noncoding RNAs. Using the novel ceRNAxis pipeline, THUMPD3‐AS1/miR‐485‐5p/ARHGAP8 is identified as a key regulator of actin cytoskeletal remodeling through noncanonical RhoB/C‐ROCK2 signaling. Modulating this axis ameliorates SCZ‐like phenotypes in mice and aligns with patient‐derived samples, highlighting a therapeutic strategy.

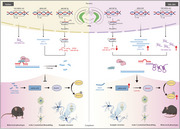

## 1 Introduction

Schizophrenia (SCZ) is a severe neuropsychiatric disorder, is characterized by cognitive deficits and impaired synaptic function.^[^ ^1^, ^2^, ^3^ ^]^ Evidence suggests that dysregulation of the cytoskeleton, particularly actin cytoskeletal remodeling mediated by Rho GTPase signaling, governs synaptic spine plasticity and stability, playing a crucial role in the pathophysiology of SCZ, which leads to synaptic instability and neuronal dysfunction.^[^ ^4^ ^]^ Notably, hippocampal dysfunction strongly correlates with cognitive deficits and impaired synaptic function in SCZ patients,^[^ ^5^, ^6^, ^7^ ^]^ correlates with aberrant Rho/ROCK activity and reduced dendritic complexity.^[^ ^8^, ^9^ ^]^ However, the upstream drivers of these abnormalities remain poorly defined. While long noncoding RNAs (lncRNAs) and microRNAs (miRNAs) have been implicated in diverse diseases,^[^ ^10^, ^11^, ^12^ ^]^ including a broad regulatory role in synaptic plasticity,^[^ ^13^, ^14^, ^15^ ^]^ their involvement in Rho‐mediated actin cytoskeletal destabilization in SCZ remains poorly understood.

LncRNAs are increasingly implicated in SCZ pathogenesis through their brain‐enriched expression and multimodal regulatory capacity, including the orchestration of synaptic, epigenetic, and posttranscriptional pathways.^[^ ^16^, ^17^, ^18^ ^]^ For instance, neuron‐specific lncRNAs such as GOMAFU, which affect synaptic plasticity by modulating ERBB4 splicing^[^ ^16^ ^]^ or repressing Crybb1 by modulating BMI1 (Crybb1 promoter region).^[^ ^19^ ^]^ Alongside antisense lncRNA‐driven epigenetic control of the SCZ risk gene CSMD1 during CNS development.^[^ ^20^ ^]^ Notably, competitive endogenous RNA (ceRNA) networks are exemplified by C2orf48A, which sequesters hsa‐miR‐20b‐5p, thereby upregulating KIF23 to influence neuronal proliferation, apoptosis, and autophagy.^[^ ^21^ ^]^ These findings highlight lncRNAs as versatile regulators bridging synaptic plasticity and neurodevelopmental pathways in SCZ.

Meanwhile, miR‐485‐5p is widely expressed and highly conserved in the brain, which has been shown to play a protective role in neurological disorders such as opioid use disorder^[^ ^22^ ^]^ and neurodegenerative diseases,^[^ ^23^, ^24^ ^]^ regulating cognitive function, synaptic plasticity,^[^ ^25^ ^]^ and neuronal apoptosis.^[^ ^26^ ^]^ Additionally, miR‐485‐5p is computationally predicted to target ARHGAP8, a Rho GTPase‐activating protein (GAP) with unexplored roles in SCZ. While ARHGAP family members such as ARHGAP10^[^ ^27^ ^]^ and ARHGAP18,^[^ ^28^ ^]^ which encode Rho GTPase‐activating proteins 10 and 18, are genetically linked to SCZ. ARHGAP family proteins modulate Rho GTPase activity to regulate actin cytoskeletal reorganization, stress fiber formation, and cell morphology.^[^ ^29^, ^30^, ^31^ ^]^ However, the effects of ARHGAP8 on Rho GTPase signaling SCZ and its post‐transcriptional regulation by noncoding RNAs (ncRNAs) remain poorly understood. While cytoskeletal dysfunction is a well‐established pathological feature of SCZ,^[^ ^32^, ^33^ ^]^ and Yoshihara's group further reinforced this link through confocal and scanning electron microscopy analysis,^[^ ^34^ ^]^ few studies have elucidated how ncRNA dysregulation contributes to synaptic structural abnormalities via Rho GTPase‐mediated signaling. This represents a critical gap in our understanding of how genetic and transcriptional risk factors converge to drive cytoskeletal destabilization in SCZ.

Here, we performed whole‐transcriptome sequencing on peripheral blood samples from six SCZ patients and matched healthy controls. We further integrated these transcriptomic data with ncRNA reference databases and SCZ GWAS datasets and identified a novel ncRNA regulatory axis (THUMPD3‐AS1/miR‐485‐5p/ARHGAP8) by our systematic ceRNA pipeline ceRNAxis. ARHGAP8 expression leads to further enhanced activation of Rho GTPase signaling, particularly the RhoB/C‐ROCK2 cascade, resulting in disruption of the actin cytoskeleton. Given that dendritic spine structure and synaptic plasticity critically depend on actin cytoskeleton function, such dysregulation is a key contributor to synaptic dysfunction in neuropsychiatric disorders.^[^ ^4^ ^]^ We demonstrated that ARHGAP8, while suppressing RhoA, paradoxically hyperactivates RhoB/C‐ROCK2 signaling, triggering actin cytoskeletal remodeling and immature dendritic spines. Crucially, rescuing this axis in the MK‐801‐induced SCZ mouse model reversed both actin cytoskeletal disruption and synaptic dysfunction, mirroring dysregulation in patient‐derived samples.

## 2 Results

### 2.1 ceRNAxis Construction and Core Axis Validation In Vitro

#### 2.1.1 Construction of Pipeline ceRNAxis

**Figure 1:**
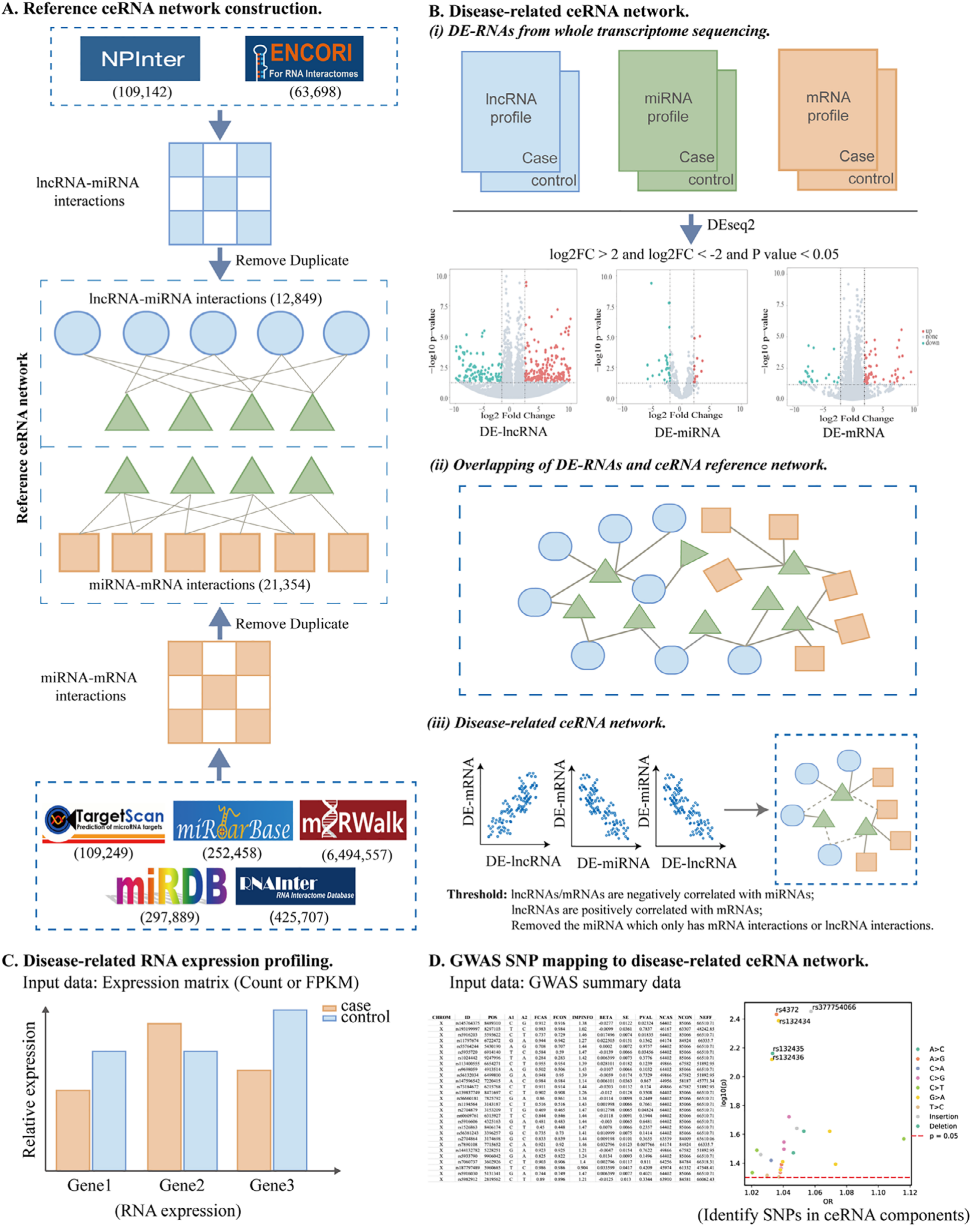
Schematic overview of the ceRNAxis pipeline. A) Establishment of the ceRNA reference database. B) Construction of lncRNA‐miRNA‐mRNA regulatory axis. C) Disease‐related expression profiling (User's own sequencing data or obtained from a database such as the GEO database, including lncRNA, miRNA, and mRNA), and it can be implemented as long as count or fpkm is available. D) GWAS SNP mapping to ceRNA axes.

In this study, we developed a systematic ceRNA pipeline (ceRNAxis) to analyze lncRNA‐miRNA‐mRNA regulatory networks. The ceRNAxis pipeline included four parts. First, we integrate multisource RNA interactions data: 1) lncRNA‐miRNA interactions: 109 142 interactions from NPInter 4.0^[^ ^35^ ^]^ and 63 698 interactions from ENCORI/starBase 2.0^[^ ^36^ ^]^; 2) miRNA‐mRNA interactions: 109 249 interactions from TargetSCAN 8.0,^[^ ^37^ ^]^ 252 458 interactions from miRTarBase 9.0,^[^ ^38^ ^]^ 6 494 557 interactions from miRWalk V3,^[^ ^39^ ^]^ 297 889 interactions from miRDB 6.0,^[^ ^40^ ^]^ and 425 707 miRNA‐mRNA or miRNA‐lncRNA interactions from RNAInter in 2020.^[^ ^41^ ^]^ After filtering, we obtained a reference ceRNA network, including 12849 lncRNA‐miRNA interactions and 21 354 miRNA‐mRNA interactions (**Figure** **1**A). Second, given the whole‐transcriptome sequencing data from a particular disease, there are three steps for the construction of the lncRNA‐miRNA‐mRNA network: 1) given the gene expression profiles from one case‐control cohort, we obtained differential expression lncRNA, miRNA, and mRNA (DE‐lncRNA, DE‐miRNA, and DE‐mRNA) by DEseq2 (|log2FC| > 2, *p* < 0.05); 2) we extracted the disease‐related network by overlapping the DE‐RNAs data with the reference ceRNA network. This step ensures that the network we constructed reflects the cohort‐specific and disease‐related gene expression changes and enhances the biological relevance of the ceRNA network; 3) Identify the ceRNA regulatory network by the criteria of lncRNA and mRNA are negatively correlated with miRNA, lncRNA/mRNA and miRNA are positively correlated, and we eliminated the miRNAs that interacted only with mRNAs or lncRNAs (Figure 1B).

Third, the disease expression profiles from specific cohorts or cell types were incorporated into ceRNAxis to show the expression of the disease‐related ceRNA network (Figure 1C). Finally, GWAS summary data from special diseases were integrated to identify genetic variants affecting components of the ceRNA network, providing an additional layer of functional annotation (Figure 1D). Taken together, this pipeline ensures reproducible and reliable discovery of regulatory pathways by using clear scoring criteria and multidatabase integration, enabling robust identification of regulatory axes. The pipeline is available at https://github.com/compbioclub/ceRNAxis. The full version of the reference ceRNA network for the ceRNAxis pipeline is available at https://zenodo.org/records/15848890.

#### 2.1.2 THUMPD3‐AS1/miR‐485‐5p/ARHGAP8 Axis Selection

**Figure 2:**
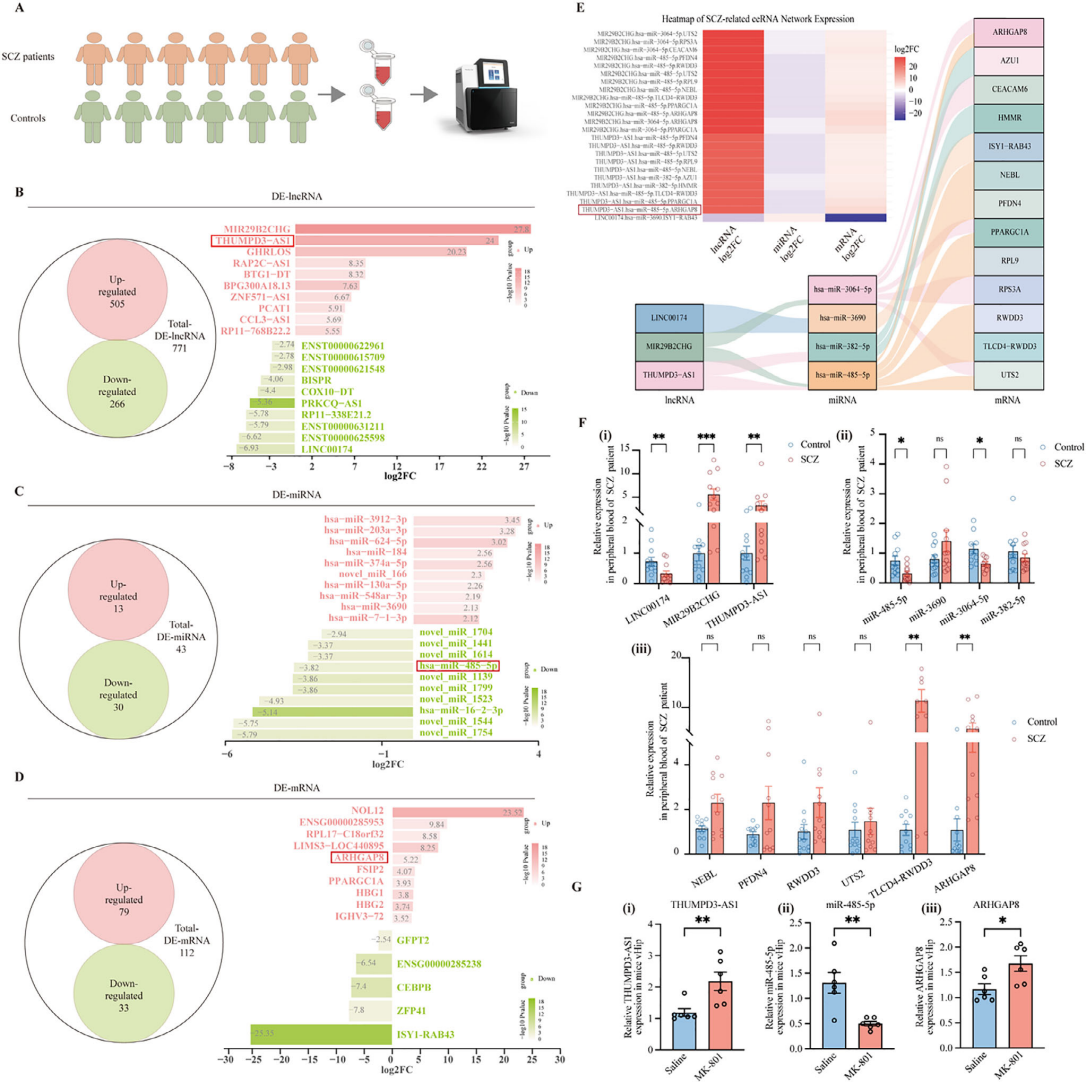
THUMPD3‐AS1/miR‐485‐5p/ARHGAP8 axis selection. A) Schematic of whole transcriptome sequencing. B–D) Venn diagrams illustrating DE‐lncRNA, DE‐miRNA, and DE‐mRNA, with the top 10 upregulated and downregulated RNAs listed. E) Construction of SCZ‐related ceRNA networks using the ceRNAxis pipeline. F) Validation of RNA expression in the peripheral blood of SCZ patients and controls by RT‐qPCR (*n* = 12 per group). G) Validation of THUMPD3‐AS1, miR‐485‐5p, and ARHGAP8 expression in vHip of MK‐801‐induced SCZ mouse by RT‐qPCR (*n* = 6 per group). GAPDH (for mRNA/lncRNA) or U6 (for miRNA) was used as the internal control in RT‐qPCR. Multiple Mann–Whitney *U* tests with FDR correction were performed. All statistical tests were two‐tailed, and data were presented as mean ± SEM. Statistical significance was defined as follows: ns (not significant, *p* ≥ 0.05); ^*^ *p* < 0.05 ^**^ *p* < 0.01, ^***^ *p* < 0.001, and ^****^ *p* < 0.0001.

We performed whole transcriptome RNA sequencing on peripheral blood samples from six SCZ patients and matched controls (**Figure** **2**A, Table S1, Supporting Information). RNA‐seq count data analysis identified distinct transcriptional profiles between groups, as demonstrated by principal component analysis (PCA) and further validated through intergroup correlation heatmap analysis (Figure S1A,B, Supporting Information), suggesting higher intragroup similarity than intergroup samples.

Using the ceRNAxis pipeline, we obtained an SCZ‐related ceRNA network from whole transcriptome sequencing count data. First, we identified 771 dysregulated lncRNAs (505 up/266 down), 43 miRNAs (13 up/30 down), and 112 mRNAs (79 up/33 down) by DESeq2 (Figure 2B–D and Table S2, Supporting Information). Bar plots listed the top 10 upregulated and downregulated DE‐lncRNA, DE‐miRNA, and DE‐mRNA (Figure 2B–D, Tables S3–S5, Supporting Information). Hierarchical clustering of these differentially expressed RNAs effectively segregated samples by disease status in expression heatmaps (Figure S1C, Supporting Information). Second, we overlapped the differentially expressed genes with the reference ceRNA network (Figure S1D, Table S6, Supporting Information). Third, we removed all positive associated miRNA‐ceRNA interactions, the miRNA and ceRNA are both upregulated or both downregulated in SCZ cases. Finally, we removed the miRNA that only has mRNA interactions or lncRNA interactions, resulting in the final SCZ‐related ceRNA network with four miRNA interactions (Figure 2E, Table S7, Supporting Information). For the benchmarking analysis, we compared ceRNA axes predicted by ceRNAxis and DLRAPom ^[^ ^42^ ^]^ in the SCZ cohort, and further examined the overlap of detected interactions in both SCZ and gestational diabetes mellitus (GDM) datasets (miRNA dataset: GSE112168,^[^ ^43^ ^]^ mRNA dataset: GSE154377)^[^ ^44^ ^]^ (Figure S2, Tables S8 and S9, Supporting Information). The results show partially overlapping yet distinct sets of ceRNA interactions across the two tools.

The THUMPD3‐AS1/miR‐485‐5p/ARHGAP8 axis was selected as the coregulatory axis through the following criteria: (1) Significant positive correlation between THUMPD3‐AS1 and ARHGAP8 expression (log2FC = 24 and 5.22, respectively), coupled with inverse regulation of miR‐485‐5p (log2FC = −3.82) in sequencing data, consistent with ceRNA network principles. (2) Genetic evidence from the CLOZUK+PGC2 GWAS summary data (Clozapine SCZ case‐control study and Psychiatric Genomics Consortium (PGC) Stage 2)^[^ ^45^ ^]^ identified 10 risk SNPs within the THUMPD3‐AS1 transcriptional region, and 9 risk SNPs within the ARHGAP8 (OR > 1, *p* < 0.05), which suggest potential involvement in the pathological processes of SCZ through posttranscriptional regulatory mechanisms (Figure S1E, Tables S10 and S11, Supporting Information). (3) Compared to the other two lncRNAs, THUMPD3‐AS1 has a relatively high sequence homology between human and mouse and is relatively highly expressed in the brain (Figure S3, Supporting Information). (4) RT‐qPCR analysis of independent blood samples (*n* = 12 per group) confirmed the dysregulation of three lncRNAs, four miRNAs, and six mRNAs (Figure 2F). (5) Biological relevance was further established through expression in the MK‐801‐induced SCZ mouse model, with axis components showing parallel alterations in the mouse vHip (*n* = 6 per group) (Figure 2G).

### 2.2 In Vitro Validation of THUMPD3‐AS1/miR‐485‐5p/ARHGAP8 Axis

#### 2.2.1 THUMPD3‐AS1 Functions as a Sponge for miR‐485‐5p in Both HEK293 and Cath.a Cell

**Figure 3:**
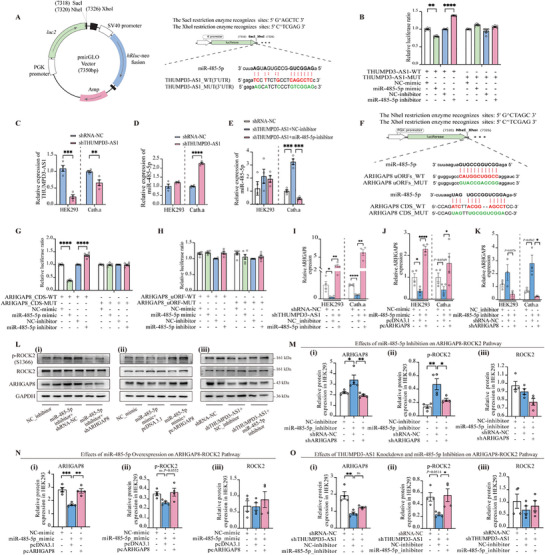
THUMPD3‐AS1 acts as a molecular sponge for miR‐485‐5p to regulate ARHGAP8 expression. A) Schematic diagram of THUMPD3‐AS1 and miR‐485‐5p binding sites construct in pmirGLO vector. B) Relative luciferase activity ratio of cells transfected with THUMPD3‐AS1(3′UTR)‐WT/MUT and miR485‐5p mimic/inhibitor. C) RT‐qPCR detected the silencing efficiency of THUMPD3‐AS1. D) Silencing of THUMPD3‐AS1 resulted in upregulation of miR‐485‐5p expression. E) Rescue experiments confirmed the regulatory relationship between THUMPD3‐AS1 and miR‐485‐5. F) Schematic sequence of the binding site of miR485‐5p targeting ARHGAP8 (uORFs and CDS). G) Relative luciferase activity ratio of cells transfected with ARHGAP8_CDS‐WT/MUT and miR485‐5p mimic/inhibitor. H) Relative luciferase activity ratio of cells transfected with ARHGAP8_uORF‐WT/MUT and miR485‐5p mimic/inhibitor. I–K) Rescue experiments confirm the regulatory relationship between miR‐485‐5 and ARHGAP8. L–O) Western Blot of ARHGAP8, p‐ROCK2 (S1366), and total ROCK2 in cell transfection with miR485‐5p mimic, inhibitor, or shTHUMPD3‐AS1. *n* = 4–6 per group. GAPDH (for mRNA/lncRNA) or U6 (for miRNA) was used as the internal control in RT‐qPCR. GAPDH was used as the loading control in Western blot. Statistical analysis was performed using one‐way ANOVA followed by Tukey's post hoc test. Data were presented as mean ± SEM. Statistical significance was defined as follows: ns (not significant, *p* ≥ 0.05); ^*^ *p* < 0.05, ^**^ *p* < 0.01, ^***^ *p* < 0.001, and ^****^ *p* < 0.0001.

To systematically validate the ceRNA interaction between THUMPD3‐AS1 and miR‐485‐5p, we conducted complementary molecular and functional analysis. First, we constructed dual‐luciferase reporter plasmids containing either the wild‐type (WT) or mutated type (MUT) sequence of THUMPD3‐AS1 at the predicted miR‐485‐5p binding site (**Figures** **3**A and S4A, Supporting Information). HEK293 cells co‐transfected with miR‐485‐5p mimics and WT plasmid constructs exhibited a significant reduction in relative luciferase activity compared to scramble controls. This effect was abolished in the MUT plasmid, confirming that the interaction between miR‐485‐5p and THUMPD3‐AS1 is sequence‐dependent (Figure 3B).

To establish functional reciprocity, we performed THUMPD3‐AS1 knockdown using shRNA in both HEK293 and Cath.a cell. RT‐qPCR revealed that THUMPD3‐AS1 expression was significantly reduced after shRNA THUMPD3‐AS1 transfection (Figure 3C). Correspondingly, THUMPD3‐AS1 decrease resulted in a significant upregulation of miR‐485‐5p expression (Figure 3D). Rescue experiments using miR‐485‐5p inhibitors abrogated this compensatory miRNA elevation (Figure 3E). These approaches provide conclusive evidence that THUMPD3‐AS1 acts as a ceRNA to buffer miR‐485‐5p activity through 3′UTR‐mediated sequestration.

#### 2.2.2 miR‐485‐5p Directly Targets ARHGAP8 Through CDS‐Specific Binding

To determine whether miR‐485‐5p directly targets ARHGAP8, we identified two highly conserved binding sites, one in the upstream open reading frame (uORF) and the other in the coding DNA sequence (CDS) of ARHGAP8 (Figure 3F). We then constructed dual‐luciferase reporter plasmids with either WT or MUT versions of the ARHGAP8_CDS binding site and the ARHGAP8_uORF binding sites (Figure S4B,C, Supporting Information). These plasmids were used in dual‐luciferase reporter assays to assess miR‐485‐5p binding. Transfection of miR‐485‐5p mimics in HEK293 cells specifically reduced the CDS regions’ WT relative luciferase activity, while MUT constructs showed resistance to suppression (Figure 3G). No significant modulation was observed at the uORF site (Figure 3H), suggesting CDS‐specific targeting.

Knockdown of THUMPD3‐AS1 in HEK293 and Cath.a cells decreased ARHGAP8 mRNA expression levels, an effect that was rescued by co‐treatment with miR‐485‐5p inhibitors (Figure 3I). Conversely, miR‐485‐5p overexpression (miR‐485‐5p mimics) suppressed ARHGAP8 expression. This effect was abolished by ARHGAP8 overexpression, achieved through co‐transfection with a pcDNA3.1‐based ARHGAP8 expression plasmid (pcARHGAP8) (Figure 3J). Specificity was confirmed through inverse experiments. miR‐485‐5p inhibition increased ARHGAP8 levels, which were nullified by inhibiting ARHGAP8 using a specific shRNA targeting ARHGAP8 (shARHGAP8) (Figure 3K), suggesting that miR‐485‐5p directly targets ARHGAP8 and inhibits ARHGAP8 expression.

#### 2.2.3 THUMPD3‐AS1/miR‐485‐5p Axis Regulates ARHGAP8‐ROCK2 Signaling

To delineate the functional consequences of miR‐485‐5p/ARHGAP8 regulation on Rho kinase signaling, we systematically analyzed ARHGAP8 protein and its downstream effector Rho kinase 2 (ROCK2) and ROCK2 phosphorylated form at Ser1366 (p‐ROCK2) through western blotting in the HEK293 cell line. Inhibition of miR‐485‐5p significantly upregulated both ARHGAP8 and p‐ROCK2 levels, whereas concurrent ARHGAP8 knockdown reversed this activation (Figure 3L(i),M), establishing ARHGAP8 as the critical mediator of ROCK2 phosphorylation. Conversely, transfection of miR‐485‐5p mimics suppressed ARHGAP8 expression without altering total ROCK2 or p‐ROCK2 levels. This suppression was rescued by ARHGAP8 overexpression, resulting in the upregulation of ARHGAP8 and subsequent reactivation of ROCK2 (Figure 3L(ii),N), confirming posttranscriptional control of ARHGAP8‐dependent pathway activation. Intriguingly, silencing THUMPD3‐AS1 led to a decrease in ARHGAP8 levels without any significant alteration in ROCK2 and p‐ROCK2. Remarkably, the simultaneous knockdown of THUMPD3‐AS1 and the inhibition of miR‐485‐5p restored p‐ROCK2 to baseline (Figure 3L(iii),O), demonstrating that THUMPD3‐AS1 exerts its regulatory effects exclusively via the miR‐485‐5p/ARHGAP8 axis.

These results indicate that miR‐485‐5p regulates ARHGAP8 protein levels, which in turn affect ROCK2 activation (Increased p‐ROCK2). In contrast, the effect of THUMPD3‐AS1 silencing appears to be limited to ARHGAP8 expression and has no direct significant downstream effect on the ROCK2 pathway, but rather exerts its effect indirectly by mediating miR‐485‐5p.

#### 2.2.4 ARHGAP8 Activates ROCK2 via a RhoA‐Independent Mechanism

**Figure 4:**
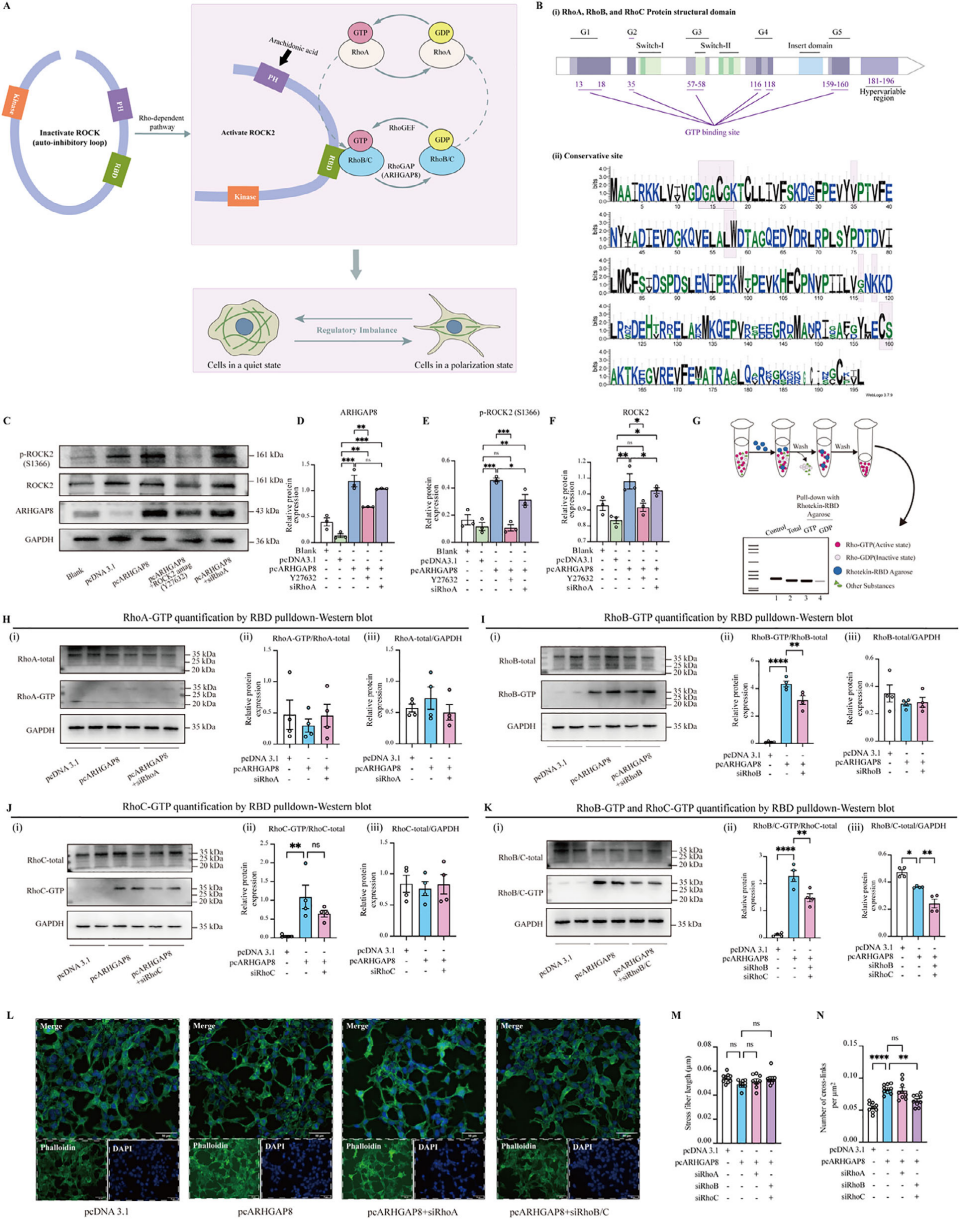
ARHGAP8 activates RhoB/C‐ROCK2 and leads to cytoskeletal disorders. A) Schematic of ARHGAP8‐mediated compensatory RhoB/C‐dependent ROCK2 activation. B) Conservation analysis of human RhoA, RhoB, and RhoC protein. (i) Domain architecture of RhoA, RhoB, and RhoC. (ii) WebLogo showed multiple sequence alignment of three proteins. The *x*‐axis is amino acid position, while the y‐axis is sequence conservation (bits) (GTP binding sites highlighted). C) Western blot of ARHGAP8, p‐ROCK2, and total ROCK2 expression. D–F) Quantification of the protein expression of ARHGAP8, p‐ROCK2, and total ROCK2. *n* = 3. G) Schematic of active Rho (GTP‐bound) enrichment via RBD‐agarose pulldown and downstream detection. H–J) Active RhoA (RhoA‐GTP), RhoB (RhoB‐GTP), RhoC (RhoC‐GTP) quantification by RBD pulldown‐Western blot. (K) Simultaneous quantification of RhoB‐GTP and RhoC‐GTP by RBD pulldown‐Western blot. *n* = 4. L) Phalloidin staining images showing stress fiber morphology in HEK293 cells. Scale bar, 50 µm. M–N) Quantification of stress fiber parameters, including fiber length and crosslinking number. GAPDH was used as the loading control in Western blot. Statistical analysis was performed using one‐way ANOVA followed by Tukey's post hoc test. Data were presented as mean ± SEM. Statistical significance was defined as follows: ns (not significant, *p* ≥ 0.05); ^*^ *p* < 0.05, ^**^ *p* < 0.01, ^***^ *p* < 0.001, and ^****^ *p* < 0.0001.

As illustrated in **Figure** **4**A, we propose a potential compensatory mechanism in which the suppression of RhoA activity by ARHGAP8 allows RhoB/C to activate ROCK2, leading to actin cytoskeletal imbalance. As a member of the RhoGAP family, ARHGAP8 regulates Rho GTPase signaling to control cytoskeletal remodeling patterns.^[^ ^31^, ^46^ ^]^ Despite sharing 85% sequence identity and conserved functional domains, Rho family members (including RhoA, RhoB, RhoC) exhibit divergent roles in cellular processes.^[^ ^47^ ^]^ Structural analysis revealed conservation in GTP‐binding domains (G1‐G5 motifs), Switch‐I/II regions, and Insert domain across Rho's three members (WebLogo bit scores≈4.0), while their C‐terminal hypervariable regions (residues 181‐196) displayed distinct sequence features (Figure 4B). Consistent with other GTPases, RhoA, RhoB, and RhoC cycle between the inactive GDP‐bound and active GTP‐bound states.^[^ ^48^ ^]^

ARHGAP8 overexpression significantly increased both ARHGAP8 expression and p‐ROCK2 levels, indicating activation of the ROCK2 pathway. Treatment with the ROCK2‐specific inhibitor Y‐27632 significantly reduced p‐ROCK2 levels, confirming the role of ROCK2 in ARHGAP8‐induced phosphorylation (Figure 4C–F). These findings support that ARHGAP8 activates ROCK2 through a RhoA‐independent mechanism.

#### 2.2.5 ARHGAP8 Activates RhoB/C‐ROCK2 and Leads to Actin Cytoskeletal Disorders

To assess the regulatory effects of ARHGAP8 on Rho family GTPase activity, we performed RhoA‐GTP, RhoB‐GTP, and RhoC‐GTP pull‐down assays (Figure 4G). ARHGAP8 overexpression did not significantly alter RhoA‐GTP levels (pcNC: 0.4703 versus pcARHGAP8: 0.2983, mean values). Similarly, RhoA knockdown in ARHGAP8‐overexpressing cells showed no effect on RhoA‐GTP levels (Figure 4H), suggesting that ARHGAP8 does not modulate RhoA activity under these conditions. In contrast, ARHGAP8 overexpression significantly increased RhoB‐GTP levels by approximately 45.1‐fold (0.0961 versus 4.3380, Figure 4I) and RhoC‐GTP levels by approximately 23.4‐fold (0.0464 versus 1.0890, Figure 4J). Combined analysis of RhoB‐GTP and RhoC‐GTP revealed a 19.5‐fold increase compared to control (0.1179 versus 2.281, Figure 4K). Knockdown of RhoB or RhoC in ARHGAP8‐overexpressing cells reduced RhoB‐GTP levels by 3.7‐fold (1.180 versus 4.338) and RhoC‐GTP levels by 1.7‐fold (0.6448 versus 1.0890). Furthermore, simultaneous knockdown of RhoB and RhoC resulted in a significant reduction of both RhoB/C GTP‐bound forms (1.480 versus 2.2810). Dual knockdown of RhoB and RhoC reduced their total protein level but paradoxically increased their GTP‐bound forms, indicating persistent activation of residual proteins. Similarly, ARHGAP8 overexpression selectively increased the active forms of RhoB/C without affecting total protein abundance, consistent with a compensatory mechanism upon RhoA suppression.

Morphological assessment in HEK293 cells revealed that ARHGAP8 overexpression induced a partial acquisition of polarized cell morphology, characterized by reduced cytoplasmic spreading and sharpened cell edges, suggestive of increased cellular contractility and aberrant polarity driven by ROCK2 hyperactivation (Figure 4L). Consistently, cytoskeletal analysis demonstrated that although ARHGAP8 overexpression did not alter the average length of individual stress fibers (Figure 4M), it significantly increased the number of cross‐linking points between fibers (Figure 4N), suggesting a shift from longitudinal fiber elongation to enhanced lateral networking. This reorganization likely contributes to impaired actin cytoskeletal plasticity and altered cellular mechanics associated with neurodevelopmental pathologies such as SCZ. Knockdown of RhoA alone in ARHGAP8‐overexpressing cells produced similar actin cytoskeletal alterations, suggesting that RhoA suppression is insufficient to block compensatory signaling. In contrast, simultaneous knockdown of RhoB and RhoC partially restored cross‐linking patterns toward normal, consistent with attenuation of ROCK2 hyperactivation.

### 2.3 Functional Validation of the THUMPD3‐AS1/miR‐485‐5p/ARHGAP8 Axis in the SCZ Mouse Model

#### 2.3.1 vHip as the Key Region for Functional Validation

To investigate the THUMPD3‐AS1/miR‐485‐5p/ARHGAP8 axis in hippocampal subregions, we compared its expression between the dorsal hippocampus (dHip) and vHip. RT‐qPCR and western blot analysis revealed that MK‐801 groups induced more pronounced dysregulation of both RNA and protein levels of this axis in the vHip compared to the dHip (Figure S5, Supporting Information). To assess neuronal activity, we quantified c‐fos expression in hippocampal subregions. The vHip showed significantly reduced c‐fos‐positive (c‐fos^+^) cells in CA1 and CA3 of the MK‐801 group, with overall higher levels in the vHip compared to the dHip (Figure S6, Supporting Information). Given the stronger statistical signal in molecular alterations, more reduction in neuronal activity, and relevance to emotion^[^ ^49^, ^50^ ^]^ and social behavior,^[^ ^51^, ^52^ ^]^ we prioritized vHip for subsequent mechanistic studies.

#### 2.3.2 miR‐485‐5p Ameliorates SCZ‐Like Phenotypes via ARHGAP8 Suppression

##### miR‐485‐5p Attenuates Anxiety‐Like Behavior

**Figure 5:**
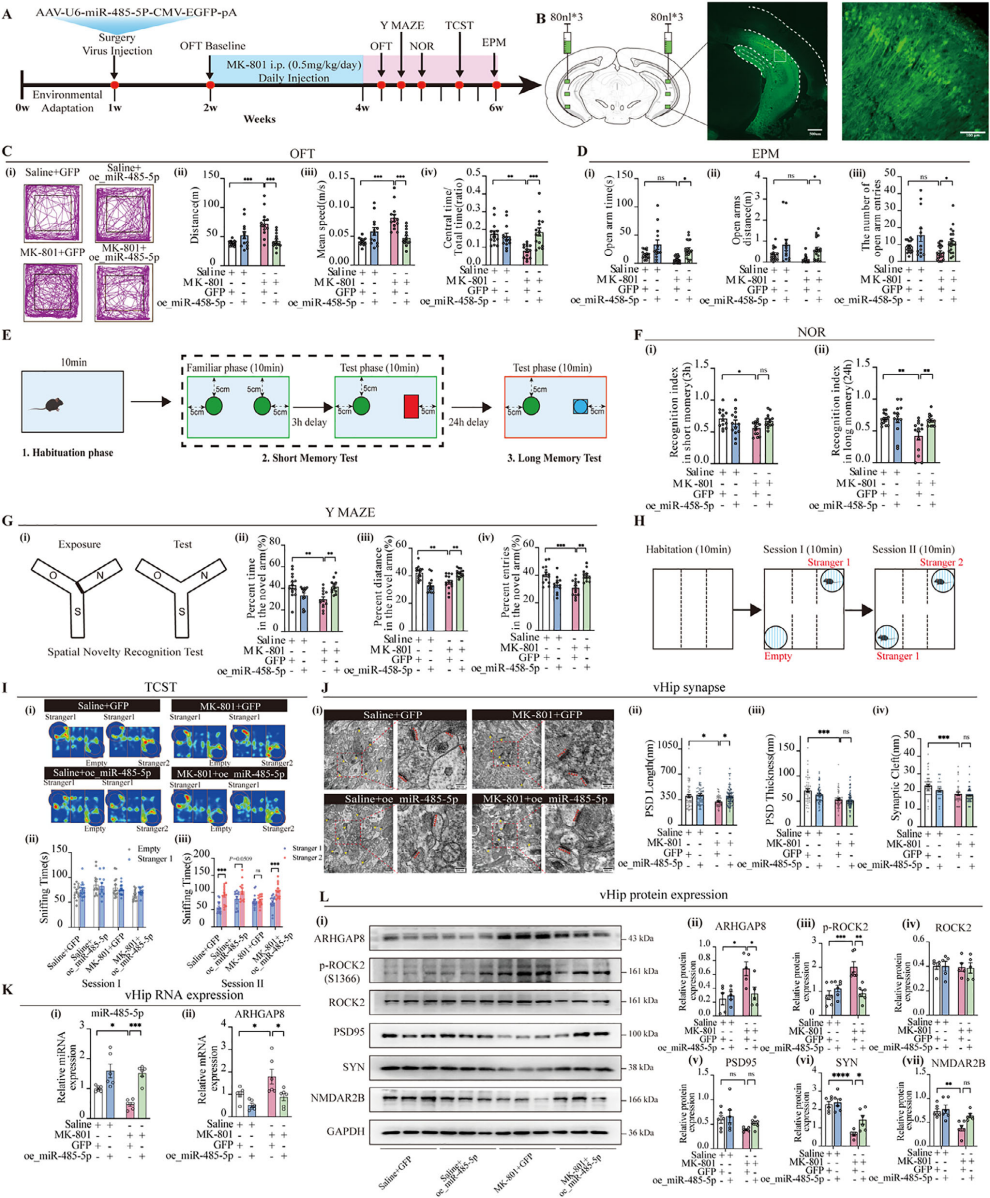
miR‐485‐5p ameliorated SCZ‐like phenotype by regulating ARHGAP8 in the MK‐801‐induced SCZ mouse model. A) Timeline of MK‐801 modeling, viral intervention, and behavioral assessments in mice. B) Representative vHip virus green fluorescent protein expression (oe‐miR‐485‐5p). C) The total distance traveled (i), mean speed (ii), and time spent in the open field test (OFT) center zone (iii) were analyzed. Representative traces in OFT (iv). *n* = 12–14 per group. D) Time spent (i), distance (ii), and number of entries (iii) in open arms were recorded in elevated plus maze (EPM), respectively. *n* = 12–14 per group. E) Diagram depicting the workflow of the novel object recognition (NOR) experiment. F) Recognition index assessment in mice with 3 h(i) or 24 h(ii) test in the NOR test. *n* = 12–14 per group. G) Diagram depicting the workflow of the Y MAZE experiment (i), percent time in the novel arm (ii), percent distance in the novel arm (iii), and percent entries in the novel arm (iv). *n* = 12–14 per group. H) Diagram depicting the workflow of the three‐chamber sociability test (TCST) experiment. I) Representative traces in TCST (i), socialization (ii), and social novelty preference (iii). *n* = 12–14 per group. J) Representative transmission electron microscopy (TEM) images of synapses of vHip neurons. The arrow indicates synapses (i). Scale bar = 500 nm. Quantitative measures of synaptic ultrastructure (ii–iv). *n* = 3 per group. K) Relative RNA expression in vHip. *n* = 5–6 per group. L) Relative protein expression in vHip. *n* = 5–6 per group. GAPDH (for mRNA/lncRNA) or U6 (for miRNA) was used as the internal control in RT‐qPCR. GAPDH was used as the loading control in the Western blot. Statistical analysis was performed using mixed RM two‐way ANOVA followed by Šidák's test. Data were presented as mean ± SEM. Statistical significance was defined as follows: ns (not significant, *p* ≥ 0.05); ^*^ *p* < 0.05, ^**^ *p* < 0.01, ^***^ *p* < 0.001, and ^****^ *p* < 0.0001.

To investigate the therapeutic potential, we stereotaxically delivered adeno‐associated virus (AAV) vectors encoding miR‐485‐5p overexpression (oe_miR‐485‐5p) into the vHip (Coordinates: Anteroposterior (AP): −3.5, Mediolateral (ML): ±3.4, Dorsoventral (DV): −4.4/−3.7/−3.1). The experimental design and efficient viral targeting are outlined in **Figure** **5**A,B. Following a 1‐week postoperative recovery period, mice received daily intraperitoneal injections of MK‐801 (0.5 mg^−1^kg^−1^day^−1^) for 14 consecutive days to induce SCZ‐like behaviors. Behavioral assessments were conducted after the final injection. Compared with saline‐treated controls, the MK‐801‐induced SCZ mouse model exhibited significant hyperlocomotion, as evidenced by increased total distance traveled and average speed, along with anxiety‐like behaviors, indicated by reduced time spent in the center zone in the open field test (OFT) (Figure 5C). Notably, overexpression of miR‐485‐5p via vHip AAV injection before MK‐801 administration effectively ameliorated these behavioral abnormalities. In the elevated plus maze (EPM), although MK‐801 administration resulted in a reduction in open arm time, open arm distance, and the number of open arm entries compared with saline‐treated controls, these differences did not reach statistical significance. However, overexpression of miR‐485‐5p significantly improved these behavioral parameters in MK‐801‐treated mice, as indicated by increased open arm time, open arm distance, and the number of open arm entries compared with the MK‐801 group (Figure 5D). These results suggest that pre‐emptive modulation of miR‐485‐5p may confer protective effects against MK‐801‐induced behavioral disturbances.

##### miR‐485‐5p Ameliorates Cognitive and Social Deficits

Following the protocol outlined in Figure 5E, we further assessed cognitive performance using the novel object recognition (NOR) test. miR‐485‐5p overexpression partially restored object memory, as indicated by increased novel object exploration time and higher recognition index at both short‐term (3 h) and long‐term (24 h) assessments, suggesting a protective effect against MK‐801‐induced cognitive impairment (Figure 5F). In the Y‐maze test, MK‐801‐induced mice exhibited impaired cognitive flexibility, as reflected by reduced time spent and distance traveled in the novel arm, and the number of entries into the novel arm. Notably, miR‐485‐5p overexpression partially restored exploratory behavior in the novel arm, indicating improved cognitive performance (Figure 5G). Social memory was assessed using the three‐chamber sociability test (TCST) (Figure 5H). MK‐801 mice showed no preference between Stranger 1 and Stranger 2, suggesting impaired social novelty recognition. In contrast, miR‐485‐5p overexpression reinstated normal sociability, evidenced by increased interaction time with Stranger 2 (Figure 5I). Together, these findings suggest that vHip overexpression of miR‐485‐5p ameliorates MK‐801‐induced deficits in cognitive flexibility and social memory associated with SCZ‐like behaviors.

##### miR‐485‐5p Alleviates Synaptic Structural Abnormality by Reducing ARHGAP8 Expression

We assessed the synaptic structural abnormality within the vHip of the MK‐801‐induced mouse model. Transmission electron microscopy (TEM) analysis demonstrated that prior overexpression of miR‐485‐5p conferred protection against MK‐801‐induced synaptic abnormalities, particularly preserving postsynaptic density (PSD) length. However, these effects did not extend to changes in PSD thickness and synaptic cleft (Figure 5J). To validate the efficacy of miR‐485‐5p overexpression and its regulatory impact on ARHGAP8, RT‐qPCR demonstrated a significant upregulation of miR‐485‐5p expression after viral overexpression, accompanied by a marked downregulation of ARHGAP8. These findings confirm successful miR‐485‐5p delivery and its suppressive effect on ARHGAP8 in vivo (Figure 5K).

Subsequently, we examined the expression of ARHGAP8 and synapse‐associated proteins (Figure 5L). A significant upregulation of ARHGAP8 expression was observed in the vHip of MK‐801 mice, which led to the activation of ROCK2. However, this activation was counteracted by overexpressing miR‐485‐5p. In addition, overexpressing miR‐485‐5p counteracted the MK‐801‐induced reduction of synapse‐associated proteins, including PSD‐95 protein (PSD95), synaptophysin (SYN), and NMDA receptor 2B (NMDAR2B). Collectively, these observations indicate that the expression of miR‐485‐5p significantly attenuates MK‐801‐induced SCZ behavior, partially restores synaptic structural abnormalities, and inhibits ARHGAP8 expression and ROCK2 phosphorylation.

#### 2.3.3 THUMPD3‐AS1 Alleviated SCZ‐Like Phenotype by miR‐485‐5p/ARHGAP8 in SCZ Mice Model

##### Inhibition of THUMPD3‐AS1 Alleviate Anxiety Behaviors

**Figure 6:**
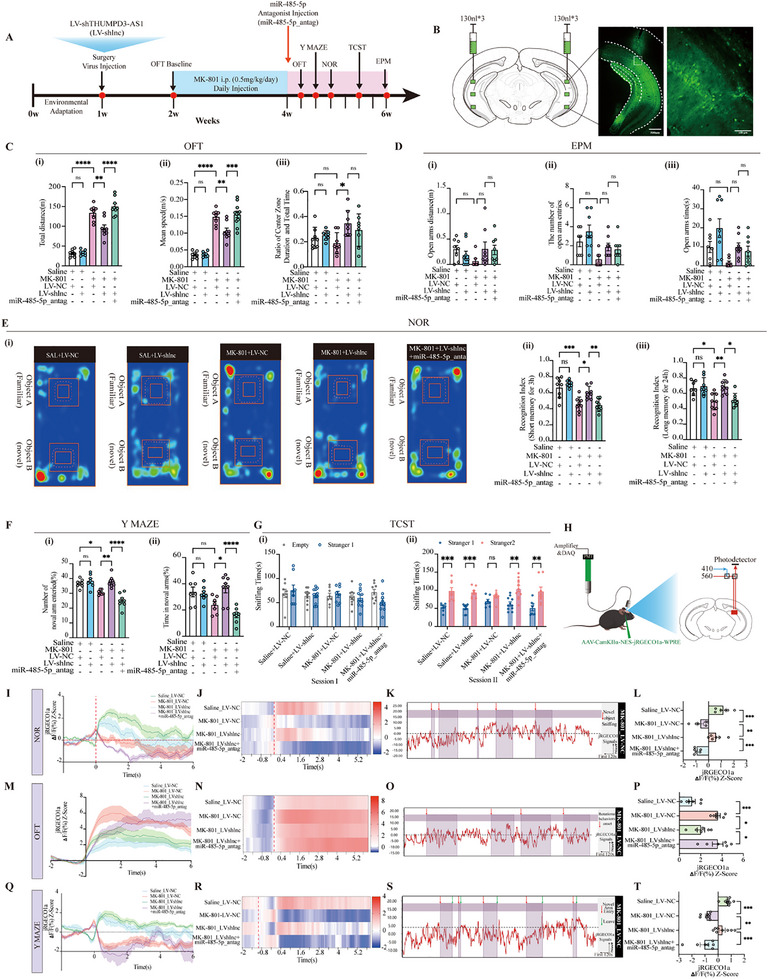
THUMPD3‐AS1 alleviated SCZ‐like phenotype by miR‐485‐5p/ARHGAP8 in the MK‐801‐induced SCZ mouse model. A) Timeline of MK‐801 modeling, viral intervention, and behavioral assessments in mice. B) Representative vHip viruses’ green fluorescent protein expression (LV‐shlnc). C) The total distance traveled (i), mean speed (ii), and time spent in the open field test (OFT) center zone (iii) were analyzed. *n* = 7–8 per group. D) Distance (i), number of entries (ii), and time spent (iii) in open arms were recorded in elevated plus maze (EPM), respectively. *n* = 7–8 per group. E) Representative traces in novel object recognition (NOR) (i), recognition index assessment in mice with 3 h (ii) or 24 h (iii) test in NOR test. *n* = 7–8 per group. F) Percent entries in the novel arm (i) and percent time in the novel arm (ii) in the Y MAZE experiment. *n* = 7–8 per group. G) Socialization (i) and social novelty preference (ii) in TCST. *n* = 7–8 per group. H) Diagram depicting the workflow of the fiber photometry. I–L) Fiber photometry to record jRGECO1a‐based calcium signals from vHip glutamatergic neurons during the NOR test (novel object sniffing). *n* = 6 per group. M–P) Fiber photometry to record jRGECO1a‐based calcium signals from vHip glutamatergic neurons during the OFT test (rotational behaviors onset). *n* = 6 per group. Q–T) Fiber photometry to record jRGECO1a‐based calcium signals from vHip glutamatergic neurons during the Y MAZE tasks test (novel arm entry). *n* = 6 per group. Statistical analysis was performed using one‐way ANOVA followed by Tukey's post hoc test. Data were presented as mean ± SEM. Statistical significance was defined as follows: ns (not significant, *p* ≥ 0.05); ^*^ *p* < 0.05, ^**^ *p* < 0.01, ^***^ *p* < 0.001, and ^****^ *p* < 0.0001.

We subsequently delivered shRNA targeting THUMPD3‐AS1 via lentiviral vectors (LV‐shTHUMPD3‐AS1, LV‐shlnc) to investigate the potential effect on SCZ‐like behavior. The detailed outline of the experimental protocol and stereotactic fluorescence imaging of lentivirus in the mouse brain is shown in **Figure** **6**A,B.

Silencing of THUMPD3‐AS1 significantly alleviated anxiety‐like behaviors in MK‐801‐induced mice, as evidenced by decreased total distance, decreased mean speed, and increased time spent in the center, effects that were reversed by miR‐485‐5p inhibition (Figure 6C). In the EPM, THUMPD3‐AS1 knockdown modestly increased open‐arm exploration and entry frequency, though the differences were not statistically significant. These effects were diminished by co‐treatment with the miR‐485‐5p antagonist (Figure 6D). These results suggest that THUMPD3‐AS1 silencing attenuates anxiety‐related behaviors via a miR‐485‐5p dependent mechanism.

##### Inhibition of THUMPD3‐AS1 Alleviate Memory Behaviors Through THUMPD3‐AS1/miR‐485‐5p

NOR testing was conducted to evaluate cognitive function. In both short‐term and long‐term assessments, THUMPD3‐AS1 knockdown significantly improved object recognition, as indicated by increased exploration time and higher recognition index for novel objects. However, these improvements were abolished by co‐administration of the miR‐485‐5p antagonist, with performance returning to levels comparable to the MK‐801 group (Figure 6E).

In the Y‐maze test, THUMPD3‐AS1 knockdown significantly improved cognitive flexibility, as evidenced by increased novel arm entries and prolonged time spent in the novel arm (Figure 6F). These improvements were abolished by the miR‐485‐5p antagonist, with behavioral performance resembling that of the MK‐801 group. In the social novelty phase of the TCST, THUMPD3‐AS1 inhibition also partially restored social preference (Figure 6G). Collectively, these findings indicate that silencing THUMPD3‐AS1 ameliorates SCZ‐like cognitive and social deficits in MK‐801‐induced mice, at least in part via a miR‐485‐5p dependent mechanism.

##### THUMPD3‐AS1 Affects the Activity of Glutamatergic Neurons by miR‐485‐5p

To further delineate the cell‐type‐specific circuit mechanisms underlying the THUMPD3‐AS1/miR‐485‐5p axis, we selectively targeted glutamatergic neurons in the vHip using a CaMKIIα promoter‐driven calcium indicator. CaMKIIα is predominantly expressed in excitatory neurons and critically regulates synaptic plasticity, a process profoundly disrupted in MK‐801‐induced SCZ‐like models. Given that our previous viral manipulations were nonselective and affected multiple neuronal subtypes, this neuron‐specific approach enabled a more precise functional assessment of glutamatergic activity. In the vHip, c‐fos^+^ cells were significantly reduced in the CA1 subregion (vCA1) of the MK‐801 group (3.6‐fold decrease, *p* < 0.0001), while vCA3 and the dentate gyrus (vDG) showed more modest decreases (Figure S6, Supporting Information). Therefore, we subsequently focused fiber photometry recordings on the vCA1, with representative images of fiber‐tip localization provided in Figure S7 (Supporting Information). We subsequently conducted fiber photometry recordings of jRGECO1a‐based calcium signals in vHip glutamatergic neurons during behavioral paradigms (NOR, OFT, and Y‐maze) to investigate how THUMPD3‐AS1 inhibition modulates activity within excitatory (Figure 6H).

In the NOR, THUMPD3‐AS1 knockdown restored calcium transients during novel object exploration in MK‐801 mice to levels comparable to the saline group, whereas co‐administration of the miR‐485‐5p antagonist attenuated this effect (Figure 6I–L). These findings indicate that suppression of THUMPD3‐AS1 enhances neuronal activity in response to object novelty via a miR‐485‐5p‐dependent mechanism. In the OFT, calcium signals recorded 6 s following the onset of stereotypic rotational behavior showed increased activity in MK‐801 mice. However, mice receiving LV‐shlnc intervention displayed calcium activity patterns closer to those of saline‐treated mice, consistent with a restoration of neuronal responsiveness. This rescue effect was diminished by miR‐485‐5p inhibition (Figure 6M–P). In the Y‐maze, compared to the MK‐801 group, THUMPD3‐AS1 knockdown increased calcium transients during novel arm exploration, further supporting the role of this lncRNA in regulating hippocampal neuron excitability. This enhancement was mitigated by miR‐485‐5p antagonism again (Figure 6Q–T).

Together, these results suggest that THUMPD3‐AS1 silencing facilitates activity‐dependent calcium signaling in vHip glutamatergic neurons through a miR‐485‐5p‐mediated pathway, potentially linking ceRNA network modulation to functional recovery at the circuit level in SCZ‐like conditions.

##### THUMPD3‐AS1 Alleviates Synaptic Structural Abnormality by miR‐485‐5p/ARHGAP8

**Figure 7:**
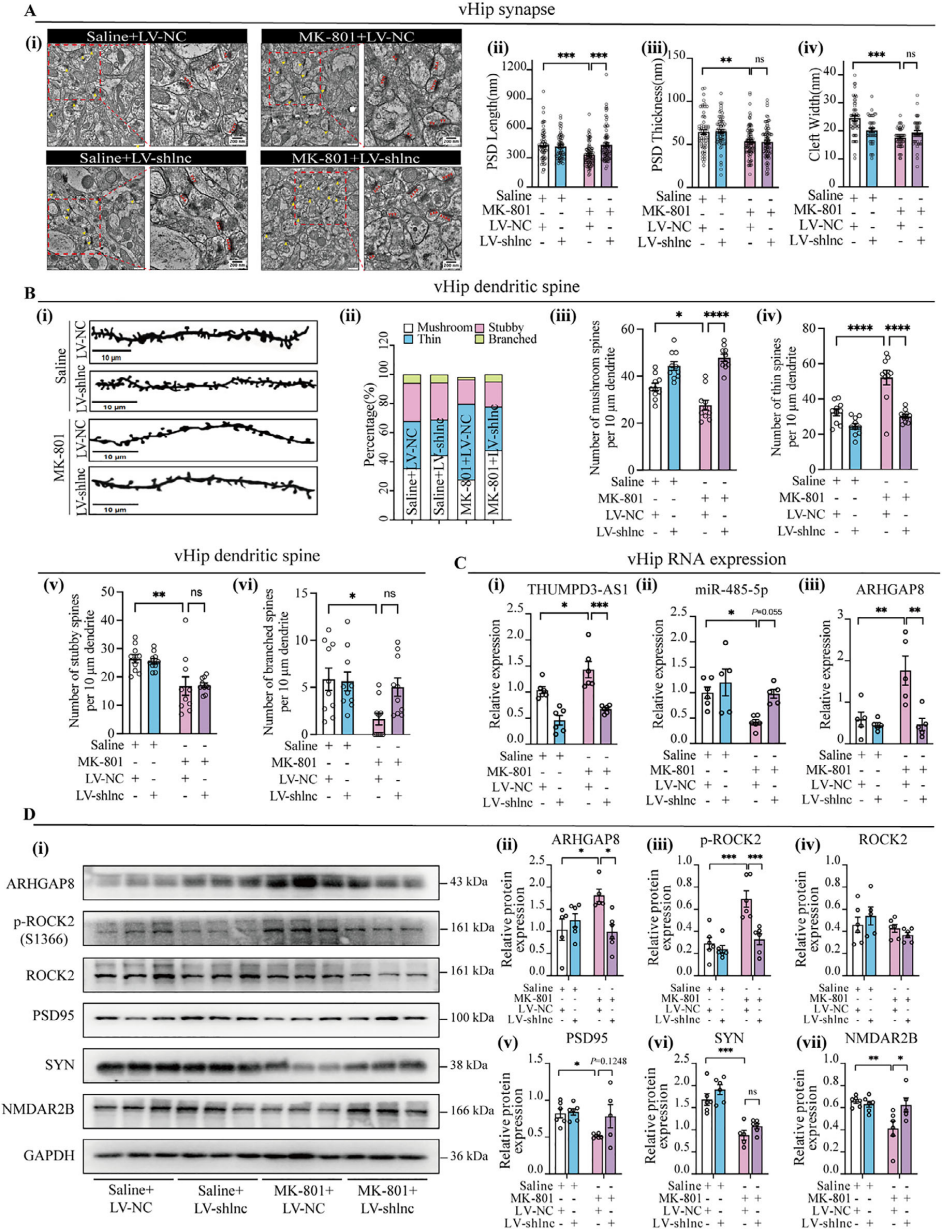
In vivo knockdown of THUMPD3‐AS1 on synaptic function in mice. A) Representative transmission electron microscopy (TEM) images of synapses of vHip neurons. The arrow indicates synapse (i). Scale bar = 500 nm. Quantitative measures of synaptic ultrastructure (ii–iv). *n* = 3 per group. B) Representative images of dendritic spines (i) and a bar graph (ii) showed the percentage of each spine type. 3 slices/sample, *n* = 3 per group. C) Relative RNA expression in vHip. *n* = 5–6 per group. D) Relative protein expression in vHip. *n* = 5–6 per group. GAPDH (for mRNA/lncRNA) or U6 (for miRNA) was used as the internal control in RT‐qPCR. GAPDH was used as the loading control in Western blot. Statistical analysis was performed using mixed RM two‐way ANOVA followed by Šidák's test. Data were presented as mean ± SEM. Statistical significance was defined as follows: ns (not significant, *p* ≥ 0.05); ^*^ *p* < 0.05, ^**^ *p* < 0.01, ^***^ *p* < 0.001, and ^****^ *p* < 0.0001.

The TEM analysis showed that THUMPD3‐AS1 knockdown partially ameliorated MK‐801‐induced synaptic ultrastructural abnormalities. Specifically, inhibition of THUMPD3‐AS1 significantly improved synaptic membrane integrity and restored PSD length. Although increases in PSD thickness and synaptic cleft width were observed, these changes did not reach statistical significance (**Figure** **7**A). Exploratory structure‐behavior correlation analysis revealed a significant association between PSD length and NOR 3 h recognition index (rho = 0.75) (Figure S8, Supporting Information). Complementary regression and slope analysis (Figure S9, Supporting Information) further supported that improvements in PSD metrics were coupled to behavioral recovery. Similarly, Golgi staining demonstrated that in the MK‐801‐induced SCZ mouse model, dendritic spine morphology exhibited a significant shift toward the immature thin spine type, accompanied by a reduction in mushroom, stubby, and branched spines. THUMPD3‐AS1 knockdown preconditioning effectively prevented the MK‐801‐induced increase in thin spines and preserved the proportion of mushroom spines. However, the reduction in stubby and branched (Figure 7B) spines was not affected by THUMPD3‐AS1 knockdown, suggesting that THUMPD3‐AS1 knockdown confers a selective protective effect on specific dendritic spine subtypes. These findings indicate that although THUMPD3‐AS1 knockdown mitigated certain MK‐801‐induced dendritic spine abnormalities, its neuroprotective effect may be limited to particular morphological categories.

**Figure 8:**
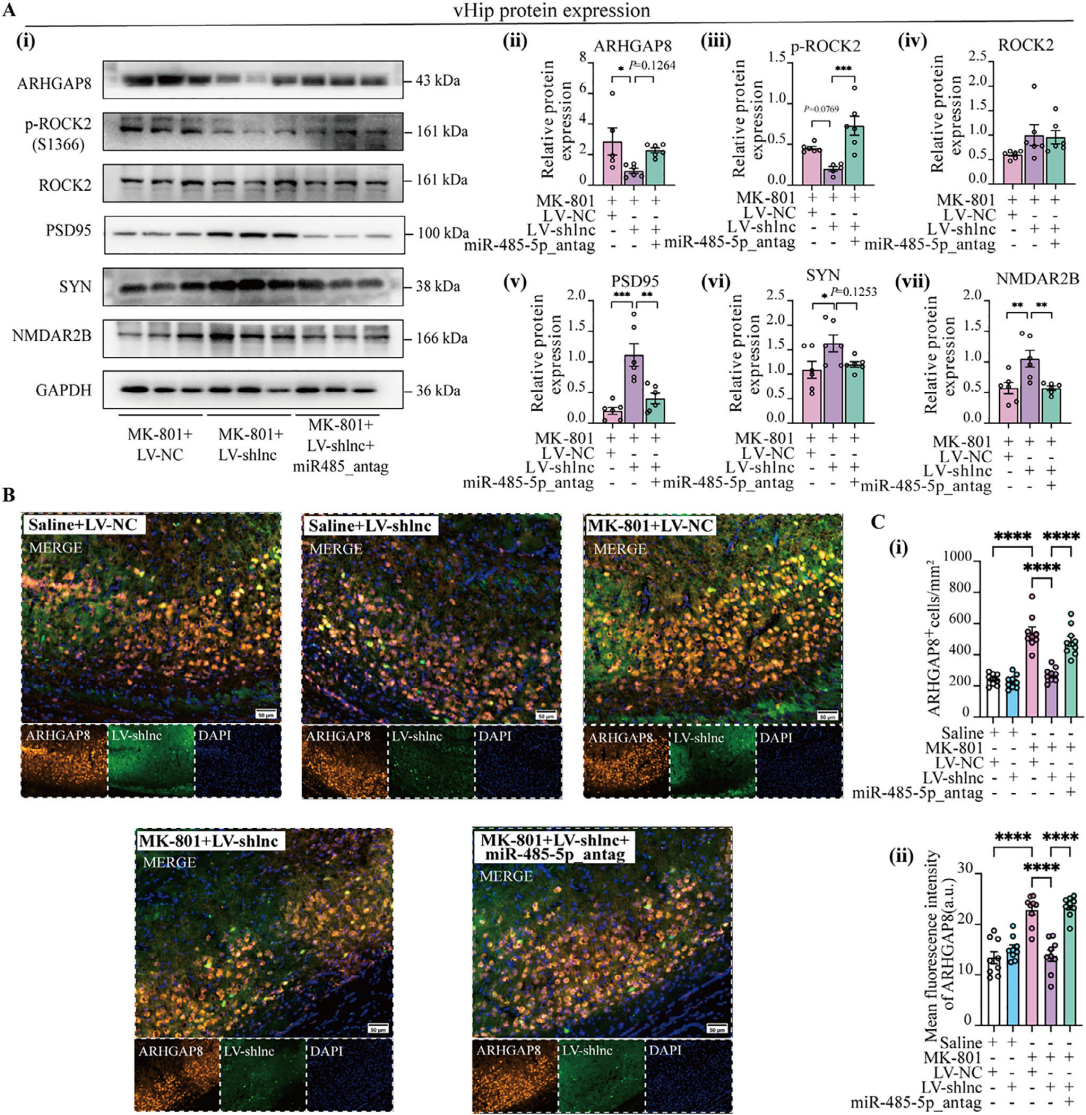
The effects of in vivo antagonism of microRNA‐485‐5p on synaptic function in mice. A) Protein expression levels were assessed following THUMPD3‐AS1 knockdown and rescue with miR‐485‐5p antagonist under MK‐801‐induced conditions. *n* = 5–6 per group. GAPDH was used as the loading control in Western blot. B,C) Immunostaining of vHip ARHGAP8 following the behavior test. Scale bar: 50 µm. Three brain slices were analyzed per mouse, with three regions imaged per slice. Data were averaged per mouse (*n* = 3 per group) and presented as mean ± SEM. Statistical analysis was performed using one‐way ANOVA followed by Tukey's post hoc test. Statistical significance was defined as follows: ns (not significant, *p* ≥ 0.05); ^*^ *p* < 0.05, ^**^ *p* < 0.01, ^***^ *p* < 0.001, and ^****^ *p* < 0.0001.

The knockdown efficiency of THUMPD3‐AS1 in the vHip tissue is shown in Figure 7C. And inhibition of THUMPD3‐AS1 reversed the MK‐801‐induced downregulation of miR‐485‐5p and the overexpression of ARHGAP8 (Figure 7C). This intervention resulted in a reduction of p‐ROCK2 and effectively mitigated the MK‐801‐induced decrease in synaptic protein levels, including PSD‐95, SYN, and NMDAR2B (Figure 7D). However, the protective effects of LV‐shlnc on synaptic integrity were partially attenuated by the miR‐485‐5p antagonist, leading to a notable recurrence of synaptic abnormalities. Western blot analysis revealed that the expression levels of synaptic‐associated proteins, ARHGAP8, ROCK2, and p‐ROCK2, were significantly elevated, approaching the abnormal levels observed in the pathological condition (**Figure** **8**A). The findings were further corroborated by immunofluorescence staining of the vHip region, which demonstrated that the miR‐485‐5p antagonist reversed the protective effects of LV‐shlnc (Figure 8B,C). These results collectively underscore the crucial role of the THUMPD3‐AS1/miR‐485‐5p axis in regulating synaptic function.

## 3 Discussion

This study elucidates a novel regulatory axis involving THUMPD3‐AS1, miR‐485‐5p, and ARHGAP8 in the pathogenesis of SCZ, linking ncRNA mediated post‐transcriptional regulation to synaptic structural abnormalities and behavioral deficits in SCZ. By integrating multilayered transcriptomic and genetic data with experimental validation in cellular and animal models, our work provides three key innovations bridging gaps in SCZ research.

### 3.1 A Multilayered Pipeline, ceRNAxis, to Decode Functional ceRNA Networks

The development of ceRNAxis addresses a critical bottleneck in prioritizing disease‐related ceRNA networks. Unlike existing tools (e.g., LncACTdb^[^ ^53^ ^]^), which rely on experimentally validated ceRNA interactions, ceRNAxis integrates negative miRNA‐ceRNA correlations, positive lncRNA‐mRNA co‐expression, GWAS risk SNP overlap, and expression level demonstration. ceRNAxis ensures a broader and more reliable interaction landscape. This multilayered filtering minimizes false positives in ceRNA predictions. This strategy identified ARHGAP8 (BPGAP1) as a hub gene associated with SCZ risk. Notably, prior GWAS findings have also linked ARHGAP8 to binge eating behavior in bipolar disorder,^[^ ^54^ ^]^ further implicating it in neuropsychiatric behavioral regulation. Meanwhile, ceRNAxis revealed ARHGAP8 as a candidate SCZ‐related gene through enrichment of 9 SNP signals (PGC GWAS summary data). Together, these features position the ceRNAxis as a robust and comprehensive platform for studying the post‐transcriptional regulatory landscape in complex diseases using whole‐transcriptome sequencing data.

ceRNAxis provides a principled framework for inferring disease‐specific ceRNA regulation by integrating high‐confidence RNA interaction data. Its two‐stage design, comprehensive network construction followed by differential expression filtering, effectively minimizes noise while preserving biological relevance, addressing key challenges in ceRNA network analysis. As an open‐source, cross‐platform tool with comprehensive documentation (https://github.com/compbioclub/ceRNAxis), ceRNAxis facilitates reproducible research in complex brain disorders. Beyond SCZ, this framework is readily extensible to other neuropsychiatric conditions and compatible with multiomics integration, enabling systematic investigation of RNA regulatory networks in disease pathogenesis.

### 3.2 ARHGAP8‐Driven RhoB/C‐ROCK2 Activation: A Noncanonical Pathway to Synaptic Pathology

Our findings redefine the role of ARHGAP8 in SCZ by uncovering its capacity to compensatorily activate RhoB/C‐ROCK2 signaling despite suppressing RhoA. The ARHGAP8 leads to decreased RhoA activity,^[^ ^31^ ^]^ while inactive RhoA will trigger a compensatory increase in RhoB activity.^[^ ^55^, ^56^ ^]^ Rho activates ROCK2 to promote actin stress fiber formation.^[^ ^57^ ^]^ The activation of ROCK2 has been shown to induce a range of biological effects, including cytoskeletal reorganization and cell motility, which is well‐documented in SCZ pathophysiology.^[^ ^9^ ^]^

This compensatory response, in which inhibition of RhoA activates RhoB/C‐ROCK2, aligns with prior studies on the crosstalk between Rho family members. RhoA‐ROCK signaling has also been implicated in oxidative stress and neuroinflammation.^[^ ^58^, ^59^ ^]^ Its aberrant activation aggravates neuroinflammatory responses, blood‐brain barrier disruption, neuronal apoptosis, and axonal damage, particularly after ischemic stroke.^[^ ^60^ ^]^ Building on this foundation, our study provides evidence supporting the involvement of ARHGAP8‐mediated RhoB/C‐ROCK2 signaling in the regulation of actin cytoskeletal remodeling. This mechanism may explain the limited efficacy of RhoA‐specific inhibitors observed in previous studies,^[^ ^61^, ^62^ ^]^ underscoring the need to consider compensatory Rho family members in therapeutic design. In particular, the different subcellular localization of RhoB/RhoC compared to RhoA may allow a spatially restricted regulation of cytoskeletal remodeling patterns.^[^ ^63^, ^64^, ^65^ ^]^ This hypothesis warrants further investigation. Our findings suggest that the observed effect is likely not due to changes in RhoB/C expression, but rather a shift in their activation states. Future studies could explore whether this is mediated by competition for upstream GEFs or feedback disruption, which may indicate a compensatory cross‐talk within the Rho GTPase family relevant to SCZ pathophysiology.

### 3.3 Dual Targeting of THUMPD3‐AS1 and miR‐485‐5p Rescues SCZ‐Like Deficits

Our findings show that both overexpression of miR‐485‐5p and knockdown of THUMPD3‐AS1 significantly improve anxiety, cognition, and social interaction behaviors in the MK‐801‐induced SCZ mouse model, highlighting the bidirectional therapeutic effects of ceRNA networks. These effects are accompanied by improved synaptic protein expression (PSD‐95, SYN, NMDAR2B) and dendritic spine morphology, suggesting that actin cytoskeletal homeostasis plays a key role in symptom amelioration. This aligns with previous studies reporting reduced PSD‐95 and synaptic proteins in SCZ brains.^[^ ^66^, ^67^, ^68^ ^]^ miR‐485‐5p, a brain‐enriched miRNA, has been implicated in synaptic plasticity^[^ ^69^ ^]^ and neurodegeneration.^[^ ^23^, ^70^ ^]^ Our finding that miR‐485‐5p directly targets ARHGAP8 aligns with studies showing that miR‐485‐5p overexpression in the hippocampus rescues synaptic deficits in epilepsy models.^[^ ^69^ ^]^ This dual‐target strategy leverages the bidirectional effect of ceRNA networks, providing a blueprint for RNA‐based therapies in SCZ and related synaptopathies.

Despite these advances, several limitations warrant consideration. First, our study utilized whole‐transcriptome sequencing on six individual pairs of samples, followed by validation in an additional 12 individual pairs. Future multicenter studies with expanded sample sizes across diverse populations are imperative to ensure reproducibility and establish clinical utility. Second, our findings from the MK‐801 model (NMDA receptor hypofunction) require validation in complementary SCZ models (maternal immune activation (MIA) model, social isolation, and DISC1 mutant models) to establish broader applicability. Third, although our rescue experiments strongly support the causal role of the THUMPD3‐AS1/miR‐485‐5p/ARHGAP8 axis, SCZ is a highly multifactorial disorder. It remains possible that other molecular pathways or circuit‐level mechanisms also contribute to the observed behavioral changes.

### 3.4 Recommendation and Future Perspectives

Our findings highlight several research priorities. First, validation of the THUMPD3‐AS1/miR‐485‐5p/ARHGAP8 axis in larger and more diverse SCZ cohorts, including postmortem brain tissue or iPSC‐derived neurons, is essential to establish clinical relevance. Second, exploring therapeutic potential through RNA‐based interventions or small molecules targeting this regulatory network warrants investigation, though optimizing delivery systems remains challenging. Third, beyond conventional antipsychotics, repurposing immune‐modulatory agents such as vitamin D may offer complementary strategies, given emerging evidence linking vitamin D to immune regulation and brain function in SCZ.^[^ ^71^, ^72^ ^]^

In conclusion, we establish the THUMPD3‐AS1/miR‐485‐5p/ARHGAP8 axis as a pivotal regulator of synaptic integrity in SCZ, mediated through RhoB/C‐ROCK2 signaling. These findings advance the understanding of ceRNA networks in neuropsychiatric disorders and propose a mechanistic connection between cytoskeletal dysregulation and behavioral abnormalities. Future studies should prioritize validation in human cohorts, development of isoform‐specific Rho GTPase inhibitors, and rational design of RNA‐based therapeutics targeting this pathway.

## 4 Experimental Section

All experimental procedures were conducted following the National Institutes of Health (NIH) Guidelines for the Care and Use of Laboratory Animals and approved by the Ethics Committee of Xi'an Jiaotong University (No. 2019‐916). The overall study workflow comprises the following four methodological modules. Detailed information on experimental materials and procedures, including human sample collection, animal housing, and statistical analysis and results, is provided in the Supplementary Materials. The primer sequences and the RNA sequences involved in this study are in Table S12 (Supporting Information).

### The Construction of the ceRNAxis Pipeline

The pipeline is divided into two major parts: establishing a reference ceRNA network and constructing the cohort/disease‐specific lncRNA‐miRNA‐mRNA network/axes. After integrating all interactions from the databases (NPInter 4.0, ENCORI/starBase 2.0, TargetSCAN 8.0, miRTarBase 9.0, miRDB 6.0, miRWalk V3, RNAInter in 2020), a reference ceRNA network was obtained, containing 12 849 lncRNA‐miRNA interactions and 21 345 miRNA‐mRNA interactions. To focus on disease‐related interactions, a subnetwork was constructed by retaining only the differentially expressed RNAs (disease group versus control group) from the ceRNA reference network. This process ensured that the interactions selected were biologically relevant. The filtered ceRNA subnetwork was subsequently analyzed to identify key regulatory axes. Third, all positively correlated miRNA‐ceRNA interactions were excluded. Next, miRNAs with only mRNA interactions or miRNA interactions were excluded, resulting in a ceRNA network with miRNA‐ceRNA interactions. Finally, SNP GWAS data related to SCZ were downloaded from CLOZUK+PGC2. The selected ceRNA axes were mapped to their genomic positions and used ceRNAxis to identify SCZ‐associated SNPs in the genes within these axes.

### THUMPD3‐AS1/miR‐485‐5p/ARHGAP8 Axis Selection and Validation

DE‐lncRNAs, DE‐miRNAs, and DE‐mRNAs were identified through whole transcriptome sequencing data of six paired SCZ samples using DESeq2 (One control sample was excluded due to the quality thresholds). Second, only differentially expressed members were retained in the reference ceRNA network. Third, all positively correlated miRNA‐ceRNA interactions were excluded. Finally, miRNAs with only mRNA or miRNA interactions were excluded, resulting in a ceRNA network with miRNA‐ceRNA interactions. A multistep validation approach was used to investigate the THUMPD3‐AS1/miR‐485‐5p/ARHGAP8 axis in SCZ. First, potential candidate regulatory axes were identified by the ceRNAxis pipeline in combination with whole transcriptome sequencing of peripheral blood RNA from SCZ patients and GWAS data from the CLOZUK+PGC2. The validation steps were further supported by RT‐qPCR analysis in both SCZ patient samples (12 pairs of SCZ cases and health controls) and the MK‐801‐induced SCZ mouse model (6 pairs of saline and MK‐801‐treated mice).

### Functional Validation of THUMPD3‐AS1/miR‐485‐5p/ARHGAP8 Validated in Cell Line

This work constructed WT and MUT vectors of THUMPD3‐AS1 and ARHGAP8, as well as miR‐485‐5p mimic and inhibitor, and performed dual luciferase reporter assays to validate their interactions in the HEK293 cell line. Lentiviral vectors (LV‐shlnc) were synthesized and transfected into HEK293 and Cath.a cell. Interference and rescue assays were conducted to elucidate the regulatory relationships between THUMPD3‐AS1 and miR‐485‐5p, and between miR‐485‐5p and ARHGAP8. To explore the role of Rho kinase signaling, ARHGAP8 in HEK293 cells were overexpressed and applied a combination of genetic perturbations (downstream RNA interference) and pharmacological inhibition (ROCK2 inhibitor). Rhotekin‐RBD agarose binding assays were conducted to assess RhoA/B/C activation, and cytoskeletal phenotyping was performed through phalloidin staining and quantitative analysis of stress fibers. This strategy allowed to examine potential compensatory ROCK2 activation through RhoB/C pathways following RhoA inhibition.

### Overexpression of miR‐485‐5p in the MK‐801‐Induced SCZ Mouse Model

To overexpress miR‐485‐5p in the vHip, mice received stereotactic microinjection of an AAV carrying oe_miR‐485‐5p. Following recovery, animals were intraperitoneally administered MK‐801 (0.5 mg^−1^kg^−1^day^−1^) or saline once daily for 14 consecutive days. Behavioral testing (OFT, EPM, NOR, Y MAZE, and TCST) was initiated on the day following the final injection. After behavioral assessments, a subset of mice underwent transcardial perfusion for TEM. The remaining mice were sacrificed by decapitation, and vHip tissues were collected for RT‐qPCR (miR‐485‐5p, ARHGAP8) and western blotting (ARHGAP8, ROCK2, p‐ROCK2, SYN, PSD‐95, NMDAR2B) to evaluate molecular changes in the miR‐485‐5p/ARHGAP8 pathway.

### Knockdown of THUMPD3‐AS1 in MK‐801‐Induced SCZ Mouse Model

To assess the functional relevance of the THUMPD3‐AS1/miR‐485‐5p/ARHGAP8 axis in SCZ‐like pathology, mice received stereotactic vHip injections of LV‐shlnc. MK‐801 or saline was administered as described above. Behavioral testing and in vivo calcium imaging via fiber photometry were performed beginning the day after the final injection. At the end of behavioral testing, mice were perfused for immunofluorescence, TEM, and Golgi staining.^[^ ^73^ ^]^ The remaining animals were sacrificed for molecular analysis. RT‐qPCR (THUMPD3‐AS1, miR‐485‐5p, ARHGAP8), western blotting, and immunofluorescence were conducted to assess transcript and protein levels of axis components and synaptic markers.

### Statistical Analysis

Data analysis and visualization were performed using R (version 4.4.1) and GraphPad Prism (version 9.0). Data normality and variance homogeneity were evaluated using the Shapiro‐Wilk and Levene's tests. When data were not normally distributed, log transformation was applied, if normality could not be achieved, nonparametric tests were used. Comparisons between two groups were performed using an unpaired t‐test for normally distributed data or the Mann–Whitney *U* test for non‐normally distributed data. For comparisons among three or more groups, one‐way ANOVA or two‐way repeated‐measures (RM) ANOVA was used. Post hoc multiple comparisons were conducted using Tukey's or Sidak's tests. All statistical tests were two‐tailed, and data were presented as mean ± standard error of the mean (SEM). Statistical significance was defined as follows: ns (not significant, *p* ≥ 0.05); ^*^ *p* < 0.05, ^**^ *p* < 0.01, ^***^ *p* < 0.001, and ^****^ *p* < 0.0001.

## Conflict of Interest

The authors declare no conflict of interest.

## Author Contributions

Z.B. conceptualized the project, secured research funding, and critically revised the manuscript. G.X.J., Z.J.Y., and G.J.Y. performed the experiments, with G.X.J. additionally responsible for data analysis, figures/tables preparation, and manuscript drafting. C.L.X. developed and implemented the analysis pipeline. G.X. is responsible for transmission electron microscopy imaging. A.N.J. contributed to the ceRNA method assessment and evaluation. Y.C.X. provided the Cath.a cell line and experimental facilities. H.Y.Y. and M.L. contributed to clinical sample collection and manuscript revision. All authors read and approved the final manuscript.

## Data and Software Availability Statement

The pipeline supporting the conclusions of this manuscript is available at https://github.com/compbioclub/ceRNAxis. A detailed tutorial and user guide are available at: https://compbioclub.github.io/ceRNAxis/, providing step‐by‐step instructions for installation, data input, and analysis workflow. The full version of the reference ceRNA network for the ceRNAxis pipeline is available at https://zenodo.org/records/15848890. All data necessary to evaluate the findings in the paper are present in the paper or supplementary materials.

## Supporting information



Supporting Information

Supporting Information

Supporting Information

Supporting Information

## Data Availability

The data that support the findings of this study are openly available in [NAME] at [DOI], reference number [REF].

## References

[1] F. G. Alvino , S. Gini , A. Minetti , M. Pagani , D. Sastre‐Yagüe , N. Barsotti , E. De Guzman , C. Schleifer , A. Stuefer , L. Kushan , C. Montani , A. Galbusera , F. Papaleo , W. R. Kates , D. Murphy , M. V. Lombardo , M. Pasqualetti , C. E. Bearden , A. Gozzi , Sci. Adv. 2025, 11, 11eadq2807. 10.1126/sciadv.adq2807 PMC11900866 40073125

[2] A. Chakraborty , N. Mukesh , A. Annamneedi , Schizophr Res 2025, 276, 106. 39883992 10.1016/j.schres.2025.01.012

[3] A. K. Manica , M. O. Daud , M. O. Faloyo , M. O. Faloyo , A. R. Akinwunmi , A. M. Adekunle , A. A. Adekola , I. A. Lawal , M. A. Salawu , J. A. Muritala , R. O. Muraina , R. A. Hassan , S. O. Ogunyemi , Neurogenetics 2025, 26, 118. 10.1007/s10048-024-00796-2 39762449

[4] W. Lei , O. F. Omotade , K. R. Myers , J. Q. Zheng , Curr. Opin. Neurobiol. 2016, 39, 86. 27138585 10.1016/j.conb.2016.04.010 PMC4987222

[5] S. K. T. Kapanaiah , C. Grimm , D. Katzel , Schizophrenia 2024, 10, 190. 10.1038/s41537-024-00513-w PMC11461789 39379378

[6] D. S. Scott , M. Subramanian , J. Yamamoto , C. A. Tamminga , Mol. Psychiatry 2024, 30, 1746. 39407000 10.1038/s41380-024-02781-5 PMC12015171

[7] L. Zhang , W. Wang , Y. Ruan , Z. Li , L. Sun , G.‐J. Ji , Y. Tian , K. Wang , BMC Psychiatry 2025, 25, 182. 40016773 10.1186/s12888-025-06632-7 PMC11866882

[8] K. A. Newell‐Litwa , M. Badoual , H. Asmussen , H. Patel , L. Whitmore , A. R. Horwitz , J. Cell Biol. 2015, 210, 2225. 10.1083/jcb.201504046 PMC4508895 26169356

[9] S. A. Swanger , A. L. Mattheyses , E. G. Gentry , J. H. Herskowitz , Cell. Logist. 2015, 5, 4e1133266. 10.1080/21592799.2015.1133266 PMC4820816 27054047

[10] M. M. Mahmoud , E. F. Sanad , R. A. A. Elshimy , N. M. Hamdy , Front. Oncol. 2021, 11, 749753. 34745973 10.3389/fonc.2021.749753 PMC8567754

[11] N. M. El‐Sheikh , A. I. Abulsoud , A. Fawzy , E. F. Wasfey , N. M. Hamdy , Pathol., Res. Pract. 2023, 24, 7154570. 10.1016/j.prp.2023.154570 37244051

[12] R. Hammad , M. A. Eldosoky , A. A. Elmadbouly , R. Hammad , M. A. Eldosoky , A. A. Elmadbouly , R. B. Aglan , S. G. AbdelHamid , S. Zaky , E. Ali , F. E.‐Z. A. E. Hakam , A. M. Mosaad , N. A. Abdelmageed , F. M. Kotb , H. G. Kotb , A. A. Hady , O. I. Abo‐Elkheir , S. Kujumdshiev , U. Sack , C. Lambert , N. M. Hamdy , J. Cancer Res. Clin. Oncol. 2023, 149, 1715349. 10.1007/s00432-023-05313-w PMC10620275 37639012

[13] A. Aksoy‐Aksel , F. Zampa , G. Schratt , Philos. Trans. R. Soc., B 2014, 369, 1652. 10.1098/rstb.2013.0515 PMC4142036 25135976

[14] E. Grinman , Y. Nakahata , Y. Avchalumov , I. Espadas , S. Swarnkar , R. Yasuda , S. V. Puthanveettil , Sci. Adv. 2021, 7, 16. 10.1126/sciadv.abf0605 PMC8051873 33863727

[15] A. H. Mohammadi , S. Seyedmoalemi , M. Moghanlou , S. A. Akhlagh , S. A. T. Zavareh , M. R. Hamblin , A. Jafari , H. Mirzaei , Mol. Neurobiol. 2022, 59, 85084. 10.1007/s12035-022-02952-x 35809155

[16] P. Teng , Y. Li , L. Ku , F. Wang , D. R. Goldsmith , Z. Wen , B. Yao , Y. Feng , Brain Behav Immun 2023, 112, 175. 37301236 10.1016/j.bbi.2023.06.009 PMC10527610

[17] M. Xie , Y. Zhang , L. Yan , M. Jin , X. Lu , Q. Yu , J. Integr. Neurosci. 2024, 23, 242. 10.31083/j.jin2302042 38419436

[18] Q. T. Zhewei Wang , H. Liao , S. Rao , X. Huang , Neurol., Psychiatry Brain Res. 2018, 30, 132.

[19] P. M. Zakutansky , Y. Feng , Cells 2022, 11, 12. 10.3390/cells11121949 PMC9221589 35741078

[20] V. M. Steen , C. Nepal , K. M. Ersland , R. Holdhus , M. Nævdal , S. M. Ratvik , S. Skrede , B. Håvik , PLoS One 2013, 8, 11e79501. 10.1371/journal.pone.0079501 PMC3828352 24244513

[21] J. Wang , Y. Liu , Y. Gao , J. Liang , B. Wang , Q. Xia , Y. Xie , F. Shan , Q. Xia , Life Sci. 2023, 312, 121205. 36410410 10.1016/j.lfs.2022.121205

[22] M. T. Zanda , G. Floris , S. E. Daws , Transl. Psychiatry 2023, 13, 1117. 10.1038/s41398-023-02423-4 PMC10082780 37031193

[23] I. S. Ryu , D. H. Kim , H.‐J. Cho , J.‐H. Ryu , Rev. Neurosci. 2023, 34, 149. 10.1515/revneuro-2022-0039 35793556

[24] S. Swarbrick , N. Wragg , S. Ghosh , A. Stolzing , Mol. Neurobiol. 2019, 56, 96156. 10.1007/s12035-019-1500-y PMC6682547 30734227

[25] J. E. Cohen , P. R. Lee , R. D. Fields , Philos. Trans. R Soc., Lond. 2014, 369, 1652. 10.1098/rstb.2013.0509 PMC4142030 25135970

[26] J. Yin , H. Chen , S. Li , S. Zhang , X. Guo , Neuromol. Med. 2021, 23, 2256. 10.1007/s12017-020-08605-3 32719988

[27] M. Sekiguchi , A. Sobue , I. Kushima , C. Wang , Y. Arioka , H. Kato , A. Kodama , H. Kubo , N. Ito , M. Sawahata , K. Hada , R. Ikeda , M. Shinno , C. Mizukoshi , K. Tsujimura , A. Yoshimi , K. Ishizuka , Y. Takasaki , H. Kimura , J. Xing , Y. Yu , M. Yamamoto , T. Okada , E. Shishido , T. Inada , M. Nakatochi , T. Takano , K. Kuroda , M. Amano , B. Aleksic , et al., Transl. Psychiatry 2020, 10, 1247. 10.1038/s41398-020-00917-z PMC7376022 32699248

[28] W. Guo , Y. Cai , H. Zhang , Y. Yang , G. Yang , X. Wang , J. Zhao , J. Lin , J. Zhu , W. Li , L. Lv , PLoS One 2017, 12, 4e0175209. 10.1371/journal.pone.0175209 PMC5383423 28384650

[29] A. Longatti , L. Ponzoni , E. Moretto , G. Giansante , N. Lattuada , M. N. Colombo , M. Francolini , M. Sala , L. Murru , M. Passafaro , Mol. Neurobiol. 2021, 58, 126092. 10.1007/s12035-021-02502-x PMC8639580 34455539

[30] M. Mori , K. Saito , Y. Ohta , PLoS One 2014, 9, 6ee100271. 10.1371/journal.pone.0100271 PMC4059726 24933155

[31] D. C. P. Wong , C. Q. Pan , S. Y. Er , T. Thivakar , T. Z. Y. Rachel , S. H. Seah , P. J. Chua , T. Jiang , T. W. Chew , P. K. Chaudhuri , S. Mukherjee , A. Salim , T. A. Aye , C. G. Koh , C. T. Lim , P. H. Tan , B. H. Bay , A. J. Ridley , B. C. Low , Mol. Biol. Cell 2023, 34, 3ar13. 10.1091/mbc.E21-03-0099 PMC10011724 36598812

[32] J. L. McGuire , E. A. Depasquale , A. J. Funk , S. M. O'Donnovan , K. Hasselfeld , S. Marwaha , J. H. Hammond , V. Hartounian , J. H. Meador‐Woodruff , J. Meller , R. E. McCullumsmith , NPJ Schizophr. 2017, 3, 30. 28900113 10.1038/s41537-017-0032-6 PMC5595970

[33] Q. Wang , N. J. Brandon , Mol. Cell. Neurosci. 2011, 48, 4359. 10.1016/j.mcn.2011.06.004 21757008

[34] S. Yoshihara , X. Jiang , M. Morikawa , T. Ogawa , S. Ichinose , H. Yabe , A. Kakita , M. Toyoshima , Y. Kunii , T. Yoshikawa , Y. Tanaka , N. Hirokawa , Cell Rep. 2021, 35, 108971. 33852848 10.1016/j.celrep.2021.108971

[35] X. Teng , X. Chen , H. Xue , Y. Tang , P. Zhang , Q. Kang , Y. Hao , R. Chen , Y. Zhao , S. He , Nucleic Acids Res. 2020, 48, D1D160. 31670377 10.1093/nar/gkz969 PMC7145607

[36] J. H. Li , S. Liu , H. Zhou , L.‐H. Qu , J.‐H. Yang , Nucleic Acids Res. 2014, 42, D92. 24297251 10.1093/nar/gkt1248 PMC3964941

[37] S. E. McGeary , K. S. Lin , C. Y. Shi , T. M. Pham , N. Bisaria , G. M. Kelley , D. P. Bartel , Science 2019, 366, 6472. 10.1126/science.aav1741 PMC7051167 31806698

[38] H. Y. Huang , Y. C. Lin , S. Cui , Y. Huang , Y. Tang , J. Xu , J. Bao , Y. Li , J. Wen , H. Zuo , W. Wang , J. Li , J. Ni , Y. Ruan , L. Li , Y. Chen , Y. Xie , Z. Zhu , X. Cai , X. Chen , L. Yao , Y. Chen , Y. Luo , S. LuXu , M. Luo , C.‐M. Chiu , K. Ma , L. Zhu , G.‐J. Cheng , C. Bai , Nucleic Acids Res. 2022, 50, D1D222. 34850920 10.1093/nar/gkab1079 PMC8728135

[39] C. Sticht , C. De La Torre , A. Parveen , N. Gretz , PLoS One 2018, 13, 10e0206239. 10.1371/journal.pone.0206239 PMC6193719 30335862

[40] Y. Chen , X. Wang , Nucleic Acids Res. 2020, 48, D1D127. 31504780 10.1093/nar/gkz757 PMC6943051

[41] Y. Lin , T. Liu , T. Cui , Z. Wang , Y. Zhang , P. Tan , Y. Huang , J. Yu , D. Wang , Nucleic Acids Res. 2020, 48, D1D189. 31906603 10.1093/nar/gkz804 PMC6943043

[42] C. Shen , H. Li , M. Li , Y. Niu , J. Liu , L. Zhu , H. Gui , W. Han , H. Wang , W. Zhang , X. Wang , X. Luo , Y. Sun , J. Yan , F. Guan , Brief Bioinform. 2022, 23, 2. 10.1093/bib/bbac046 PMC8921741 35224615

[43] S. Nair , N. Jayabalan , D. Guanzon , C. Palma , K. Scholz‐Romero , O. Elfeky , F. Zuñiga , V. Ormazabal , E. Diaz , G. E. Rice , G. Duncombe , T. Jansson , H. . D. McIntyre , M. Lappas , C. Salomon , Clin. Sci. 2018, 132, 222451. 10.1042/CS20180487 30254065

[44] G. Del Vecchio , Q. Li , W. Li , S. Thamotharan , A. Tosevska , M. Morselli , K. Sung , C. Janzen , X. Zhou , M. Pellegrini , S. U. Devaskar , Epigenetics 2021, 16, 6642. 10.1080/15592294.2020.1816774 PMC8143248 33045922

[45] A. F. Pardiñas , P. Holmans , A. J. Pocklington , V. Escott‐Price , S. Ripke , N. Carrera , S. E. Legge , S. Bishop , D. Cameron , M. L. Hamshere , J. Han , L. Hubbard , A. Lynham , K. Mantripragada , E. Rees , J. H. MacCabe , S. A. McCarroll , B. T. Baune , G. Breen , E. M. Byrne , U. Dannlowski , T. C. Eley , C. Hayward , N. G. Martin , A. M. McIntosh , R. Plomin , D. J. Porteous , N. R. Wray , A. Caballero , D. H. Geschwind , et al., Nat. Genet. 2018, 50, 3381.

[46] K. Zaoui , S. Duhamel , Bio Protoc 2020, 10, 9e3609. 10.21769/BioProtoc.3609 PMC7842779 33659574

[47] A. P. Wheeler , A. J. Ridley , Exp. Cell Res. 2004, 301, 143. 10.1016/j.yexcr.2004.08.012 15501444

[48] A. J. Ridley , J. Microsc. 2013, 251, 3242. 10.1111/jmi.12025 23488932

[49] C. Barkus , S. B. McHugh , R. Sprengel , P. H. Seeburg , J. N. P. Rawlins , D. M. Bannerman , Eur. J. Pharmacol. 2010, 626, 149. 10.1016/j.ejphar.2009.10.014 PMC2824088 19836379

[50] P. Farzamfard , A. Rezayof , S. Alijanpour , Neurosci. Lett. 2022, 780, 136649. 35461976 10.1016/j.neulet.2022.136649

[51] C. H. Chang , C. L. Su , P. W. Gean , Neuropharmacology 2018, 143, 95. 30243915 10.1016/j.neuropharm.2018.09.019

[52] I. Zoicas , J. Kornhuber , Int. J. Mol. Sci. 2019, 20, 22. 10.3390/ijms20225599 PMC6887971 31717513

[53] P. Wang , Q. Guo , Y. Qi , Y. Hao , Y. Gao , H. Zhi , Y. Zhang , Y. Sun , Y. Zhang , M. Xin , Y. Zhang , S. Ning , X. Li , Nucleic Acids Res. 2022, 50, D1D183. 34850125 10.1093/nar/gkab1092 PMC8728196

[54] S. L. McElroy , S. J. Winham , A. B. Cuellar‐Barboza , C. L. Colby , A. M.‐C. Ho , H. Sicotte , B. R. Larrabee , S. Crow , M. A. Frye , J. M. Biernacka , Transl. Psychiatry 2018, 8, 140. 29391396 10.1038/s41398-017-0085-3 PMC5804024

[55] T. T. G. Ho , S. D. Merajver , C. M. Lapière , B. V. Nusgens , C. F. Deroanne , J. Biol. Chem. 2008, 283, 3121588. 10.1074/jbc.M710033200 18524772

[56] X. Zhou , Y. Zheng , J. Biol. Chem. 2013, 288, 5136179. 10.1074/jbc.R113.515486 PMC3868732 24202176

[57] A. Pranatharthi , P. Thomas , A. H. Udayashankar , C. Bhavani , S. B. Suresh , S. Krishna , J. Thatte , N. Srikantia , C. R. Ross , S. Srivastava , J. Exp. Clin. Cancer Res. 2019, 38, 1392. 10.1186/s13046-019-1385-7 PMC6729006 31488179

[58] H. M. Al‐kuraishy , G. M. Sulaiman , H. A. Mohammed , A. I. Al‐Gareeb , A. K. Albuhadily , S. G. Mohammed , Behav. Brain Res. 2025, 484, 115524. 40043855 10.1016/j.bbr.2025.115524

[59] R. Cai , Y. Wang , Z. Huang , Q. Zou , Y. Pu , C. Yu , Z. Cai , Behav. Brain Res. 2021, 414, 113481. 34302876 10.1016/j.bbr.2021.113481

[60] T. Kimura , Y. Horikoshi , C. Kuriyagawa , Y. Niiyama , Int. J. Mol. Sci. 2021, 22, 21. 10.3390/ijms222111573 PMC8584200 34769004

[61] S. Fujimoto , M. N. Leiwe , S. Aihara , R. Sakaguchi , Y. Muroyama , R. Kobayakawa , K. Kobayakawa , T. Saito , T. Imai , Dev. Cell 2023, 58, 141221. 10.1016/j.devcel.2023.05.004 37290446

[62] S. Stern , B. J. Hilton , E. R. Burnside , S. Dupraz , E. E. Handley , J. M. Gonyer , C. Brakebusch , F. Bradke , Neuron 2021, 109, 3436. 34508667 10.1016/j.neuron.2021.08.014

[63] R. Eckenstaler , M. Hauke , R. A. Benndorf , Biochem. Pharmacol. 2022, 206, 115321. 36306821 10.1016/j.bcp.2022.115321

[64] M. A. Olayioye , B. Noll , A. Hausser , Cells 2019, 8, 12. 10.3390/cells8121478 PMC6952795 31766364

[65] A. Schaefer , N. R. Reinhard , P. L. Hordijk , Small GTPases 2014, 5, 26. 10.4161/21541248.2014.968004 PMC4601309 25483298

[66] A. J. Funk , C. A. Mielnik , R. Koene , E. Newburn , A. J. Ramsey , B. K. Lipska , R. E. McCullumsmith , Schizophr. Bull. 2017, 43, 4891. 10.1093/schbul/sbw173 PMC5472126 28126896

[67] E. P. McEachern , A. A. Coley , S.‐S. Yang , W.‐J. Gao , Neuropharmacology 2020, 179, 108277. 32818520 10.1016/j.neuropharm.2020.108277 PMC7572776

[68] P. Yang , S. Huang , Z. Luo , S. Zhou , C. Zhang , Y. Zhu , J. Yang , L. Li , Biomed. Pharmacother. 2024, 172, 116267. 38364739 10.1016/j.biopha.2024.116267

[69] K. Wang , J. Wu , J. Wang , K. Jiang , Brain Behav. 2021, 11, 8e2247. 10.1002/brb3.2247 PMC8413801 34291586

[70] S. Y. Goh , Y. X. Chao , S. T. Dheen , E.‐K. Tan , S. S.‐W. Tay , Int. J. Mol. Sci. 2019, 20, 22. 10.3390/ijms20225649 PMC6888719 31718095

[71] Y. C. Chen , Y. F. Chiang , Y. J. Lin , K.‐C. Huang , H.‐Y. Chen , N. M. Hamdy , T.‐C. Huang , H.‐Y. Chang , T.‐M. Shieh , Y.‐J. Huang , S.‐M. Hsia , Nutrients 2023, 15, 13. 10.3390/nu15132830 PMC10343446 37447156

[72] X. Cui , J. J. McGrath , T. H. J. Burne , D. W. Eyles , Mol. Psychiatry 2021, 26, 72708. 10.1038/s41380-021-01025-0 PMC8505257 33500553

[73] S. Zaqout , A. M. Kaindl , Front. Neuroanat. 2016, 10, 38. 27065817 10.3389/fnana.2016.00038 PMC4814522

