# Supplementary material for: LncRNA THUMPD3‐AS1 Regulates Behavioral and Synaptic Structural Abnormalities in Schizophrenia via miR‐485‐5p and ARHGAP8
Source: Adv Sci (Weinh). 2025 Oct 30;13(3):e08867. doi: 10.1002/advs.202508867 (PMC12806223; doi:10.1002/advs.202508867)
Supplement: Supplementary file 1 — Supporting Information [file ADVS-13-e08867-s004.docx]

**Supporting Information**

**LncRNA THUMPD3-AS1 Regulates Behavioral and Synaptic Structural Abnormalities in Schizophrenia via miR-485-5p and ARHGAP8**

*Xiaojuan Gong, Lingxi Chen, Xin Guo, Anna Jiang, Yayi He, Chunxia Yan, Liang Ma, Jiayang Gao, Jinyu Zhang, Bao Zhang^*^*

**Supplementary Figures (Figure S1-Figure S9)**

**Supplementary-Detailed Methods**

**Supplementary Tables (Table S1-Table S12)**

**Supplementary-Statistical results**

**Supplementary-PANSS**

**Supplementary Figures**

**
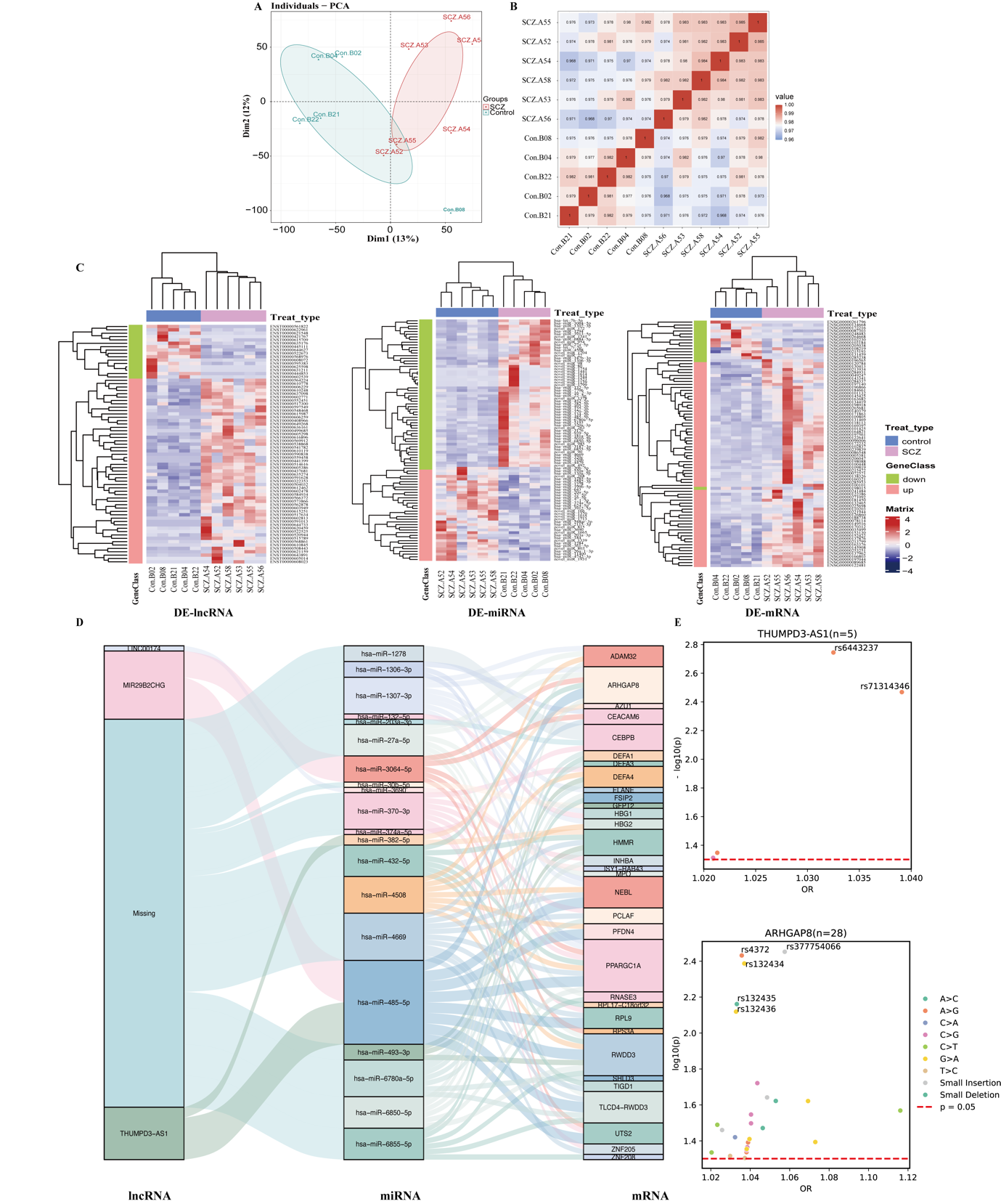
**

**Figure S1. Overview of transcriptomic profiles and preliminary construction of ceRNA networks in SCZ.** (A) PCA plot displaying sample distribution and separation between groups. (B) Correlation heatmap illustrating inter-sample expression similarity. (C) Hierarchical clustering heatmaps of differentially expressed lncRNAs, miRNAs, and mRNAs. (D) Preliminary ceRNA network constructed using the ceRNAxis pipeline. (E) Identification of SCZ-associated genetic variants in the context of the THUMPD3-AS1/miR-485-5p/ARHGAP8 regulatory axis.

**
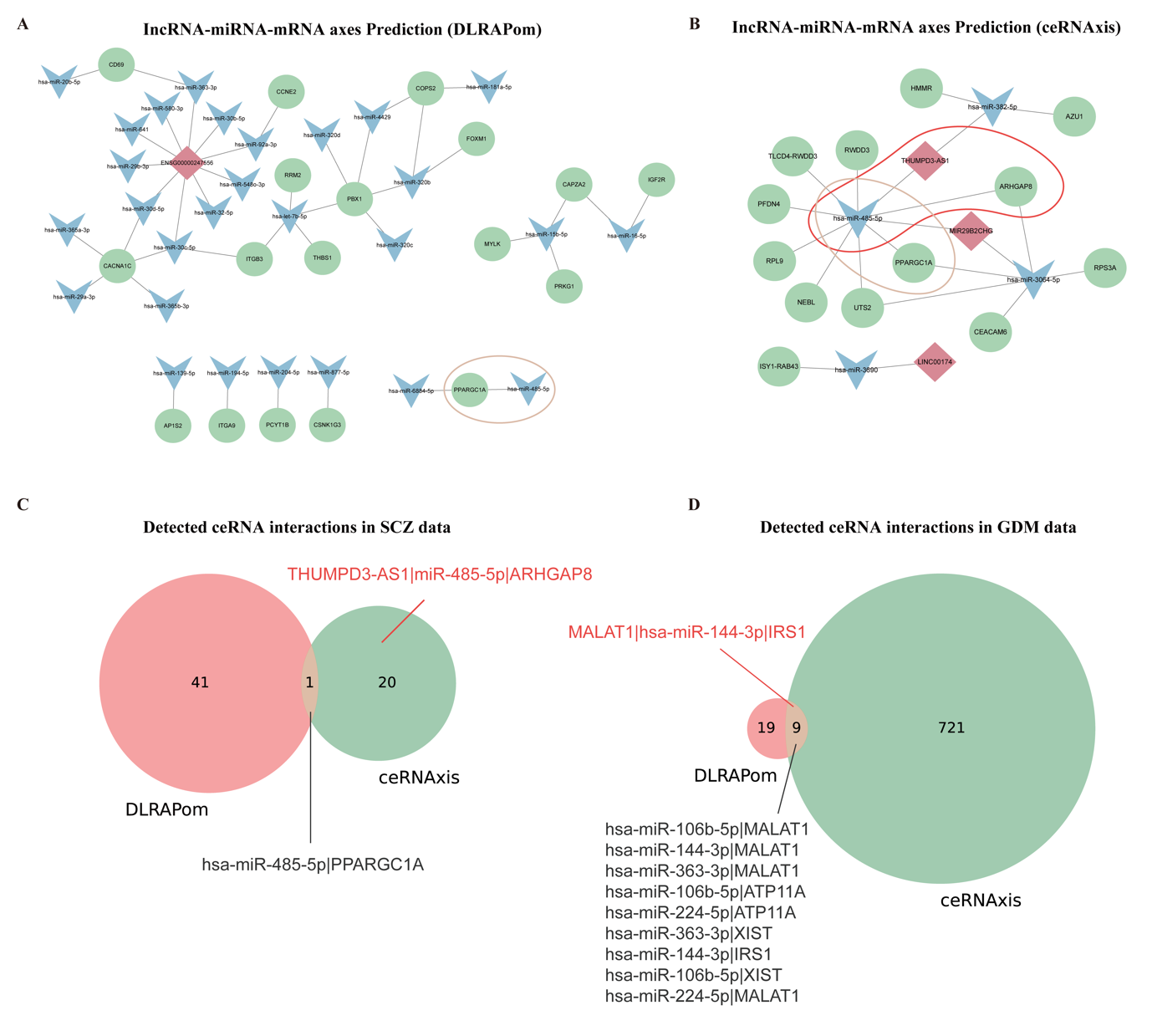
**

**Figure S2. Benchmarking ceRNAxis against DLRAPom.** (A-B) ceRNA axes predicted by DLRAPom (A) and ceRNAxis (B) in the SCZ cohort. (C-D) Venn diagrams illustrating the overlap of ceRNA interactions detected by DLRAPom and ceRNAxis in the SCZ (C) and GDM (D) cohorts, respectively. SCZ: schizophrenia; GDM: gestational diabetes mellitus.

**
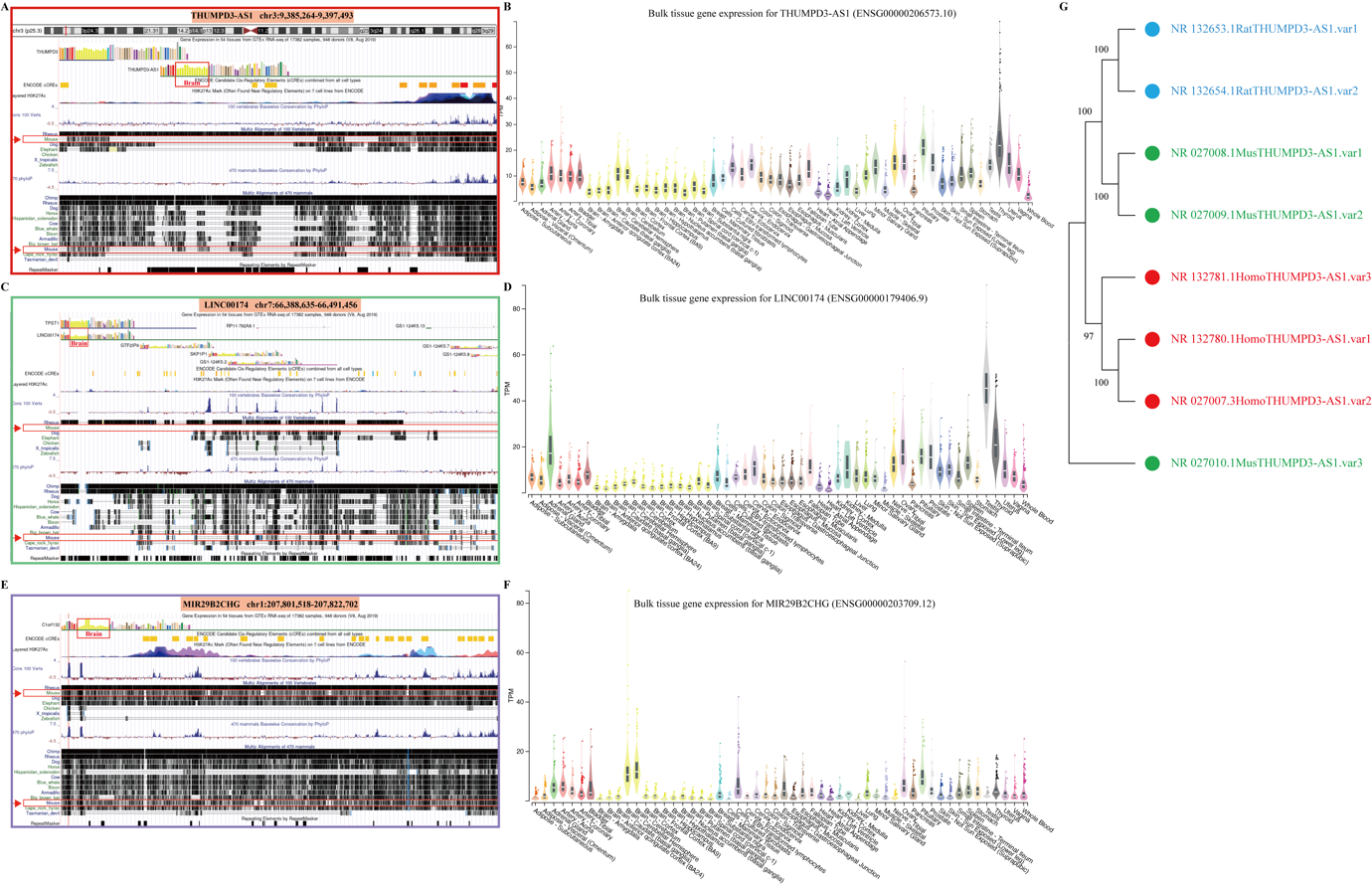
**

**Figure S3. Cross-species conservation and tissue-specific expression of candidate ceRNA axis components.** (A, C, E) UCSC Genome Browser snapshots showing genomic features, evolutionary conservation, and transcript isoforms of THUMPD3-AS1, LINC00974, and MIR485HG in the human genome (hg38), including sequence conservation across vertebrates and annotated SNPs. Red boxes highlight conserved regions. (B, D, F) Bulk tissue expression profiles of THUMPD3-AS1, LINC00974, and MIR485HG across 54 human tissues from GTEx v8. (G) Phylogenetic tree constructed using the Neighbor-Joining method based on p-distance, demonstrating sequence similarity among THUMPD3-AS1 transcripts from human (Homo sapiens), mouse (Mus musculus), and rat (Rattus norvegicus).


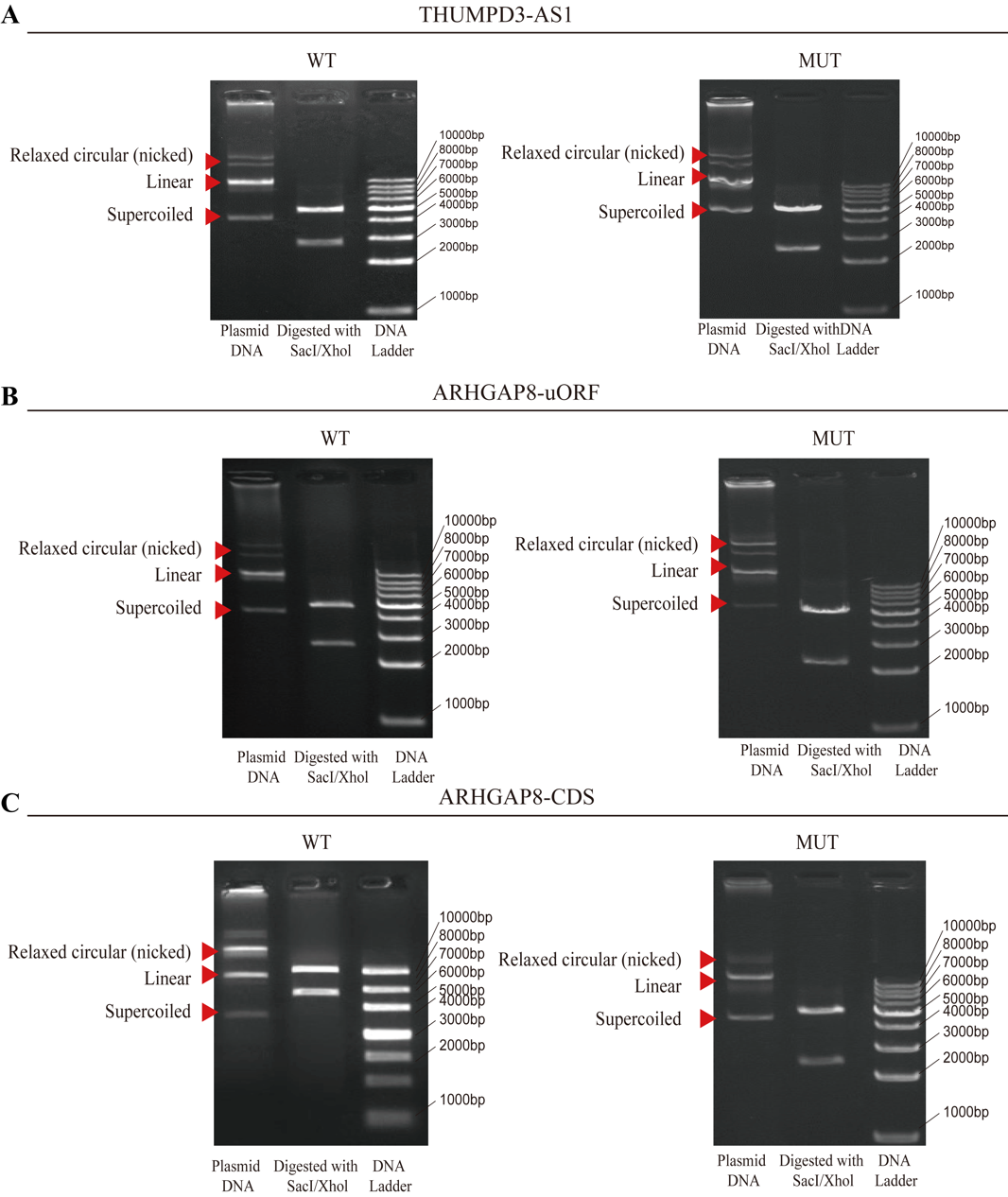


**Figure S4. THUMPD3-AS1 acts as a molecular sponge for miR-485-5p to regulate ARHGAP8 expression.** (A) Agarose gel electrophoresis results of THUMPD3-AS1 WT and MUT plasmids. (B) Agarose gel electrophoresis results of WT and MUT plasmids of the uORF regions of ARHGAP8. (C) Agarose gel electrophoresis results of WT and MUT plasmids of the CDS regions of ARHGAP8.


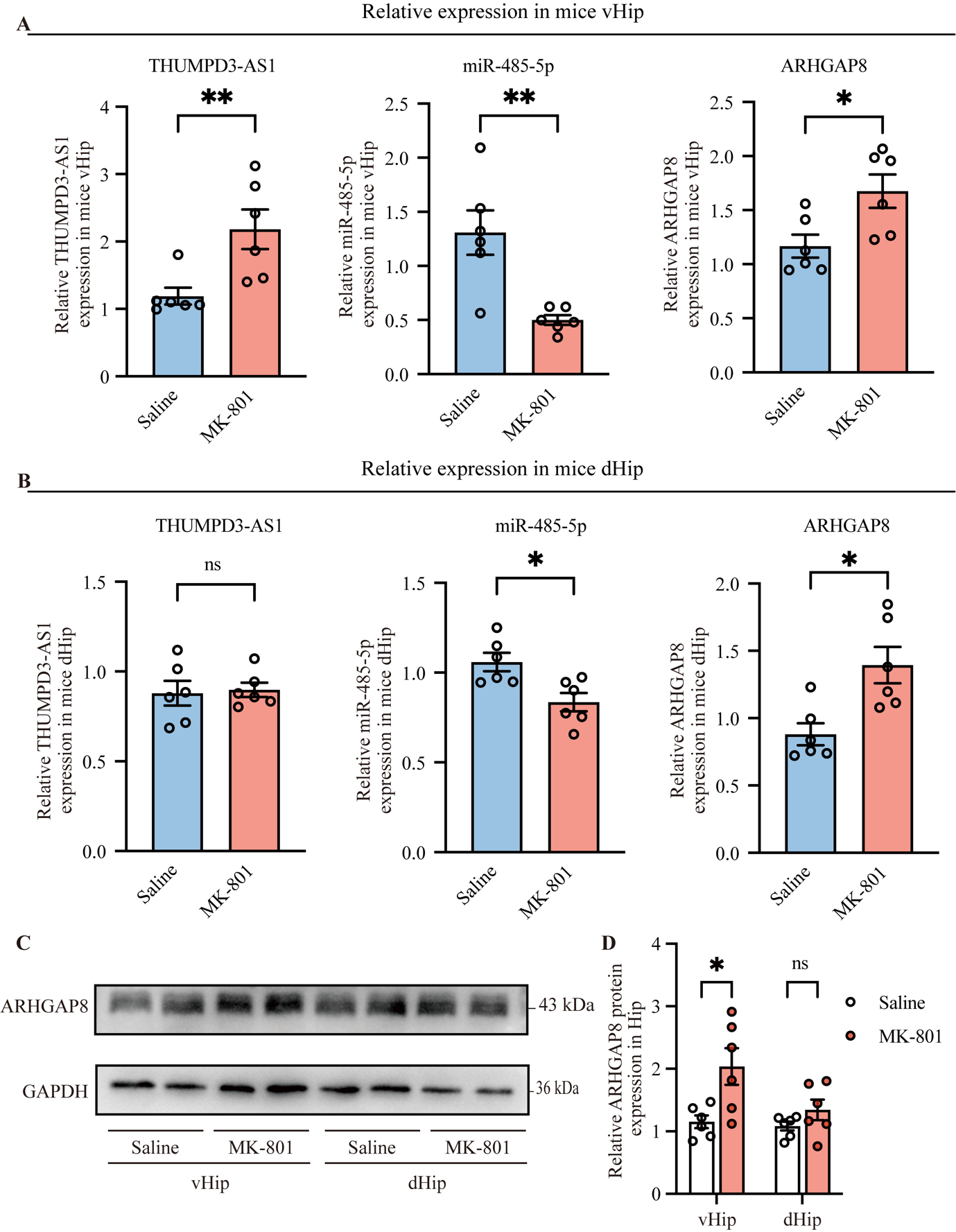


**Figure S5. Expression of the THUMPD3-AS1/miR-485-5p/ARHGAP8 regulatory axis in the vHip and dHip of mice.** (A-B) RNA expression of the THUMPD3-AS1/miR-485-5p/ARHGAP8 regulatory axis in the vHip and dHip. (C-D) Relative ARHGAP8 protein expression in vHip and dHip. GAPDH (for mRNAs/lncRNAs) or U6 (for miRNA) was used as the internal reference. n = 6 per group for dHip and n = 6 per group for vHip. Multiple Mann-Whitney U tests with FDR correction were performed. Data were presented as mean±SEM. Statistical significance was defined as follows: ns (not significant, p ≥ 0.05); p < 0.05 (*), p < 0.01 (**), p < 0.001 (***), and p < 0.0001 (****).


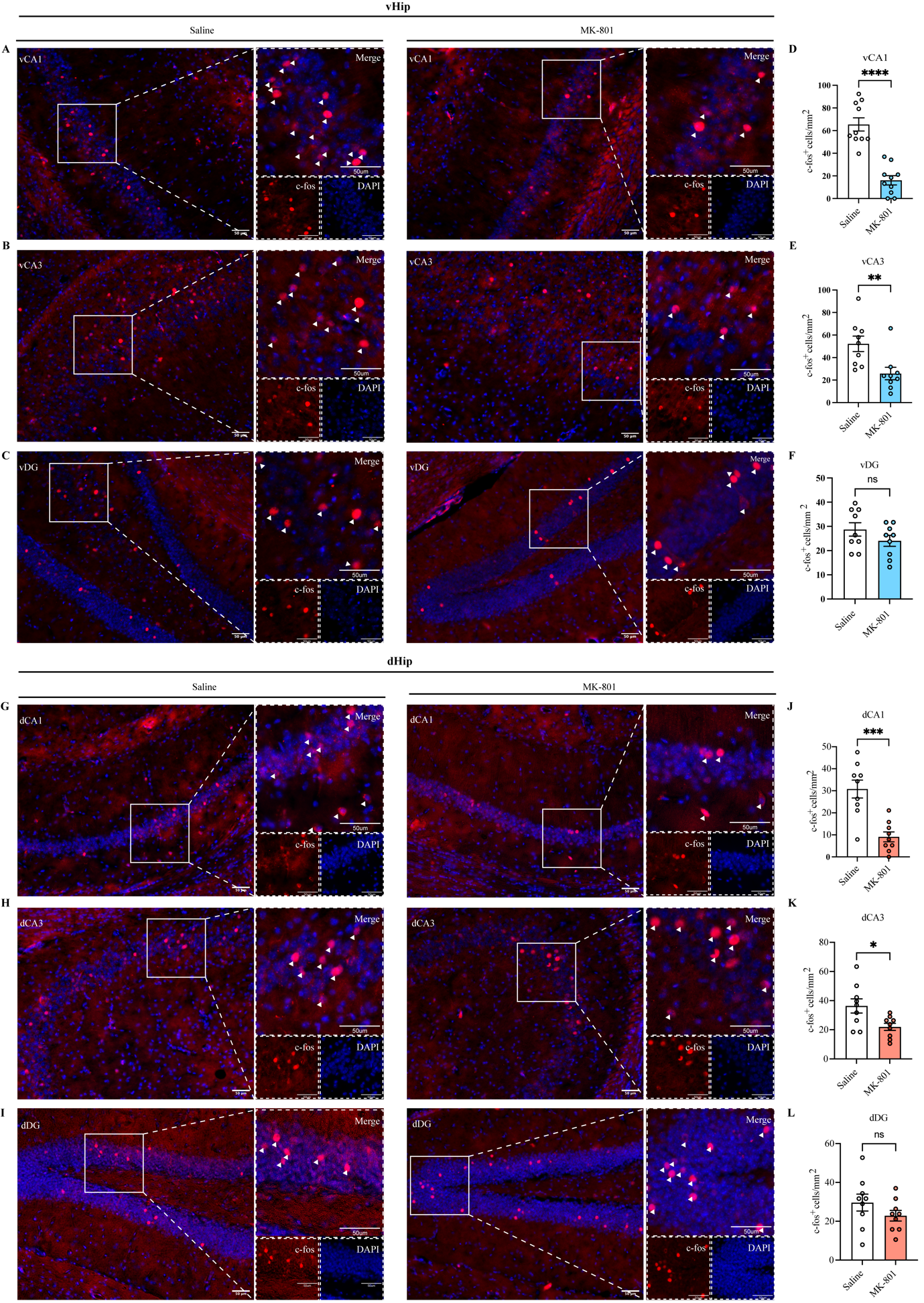


**Figure S6. Chronic MK-801 treatment reduces neuronal activity in vHip subregions.** (A-C) Representative immunofluorescence images of c-fos staining in the vCA1, vCA3, and vDG from saline-treated control mice and mice subjected to chronic MK-801 administration (0.5 mg/kg/day, intraperitoneally, for 14 consecutive days). (D-F) Quantification of c-fos^+^ cells in vHip each subregion. (G-I) Representative immunofluorescence images of c-fos staining in the dCA1, dCA3, and dDG from saline and MK-801 group. (J-L) Quantification of c-fos^+^ cells in dHip each subregion. Chronic MK-801 treatment significantly decreased c-fos expression compared with the saline group. Three brain slices were analyzed per mouse, with three regions imaged per slice. Data were averaged per mouse (n = 3 mice per group) and presented as mean ± SEM. Multiple Mann-Whitney U tests with FDR correction were performed. Scale bar = 50 μm. Statistical significance was defined as follows: ns (not significant, p ≥ 0.05); p < 0.05 (*), p < 0.01 (**), p < 0.001 (***), and p < 0.0001 (****).


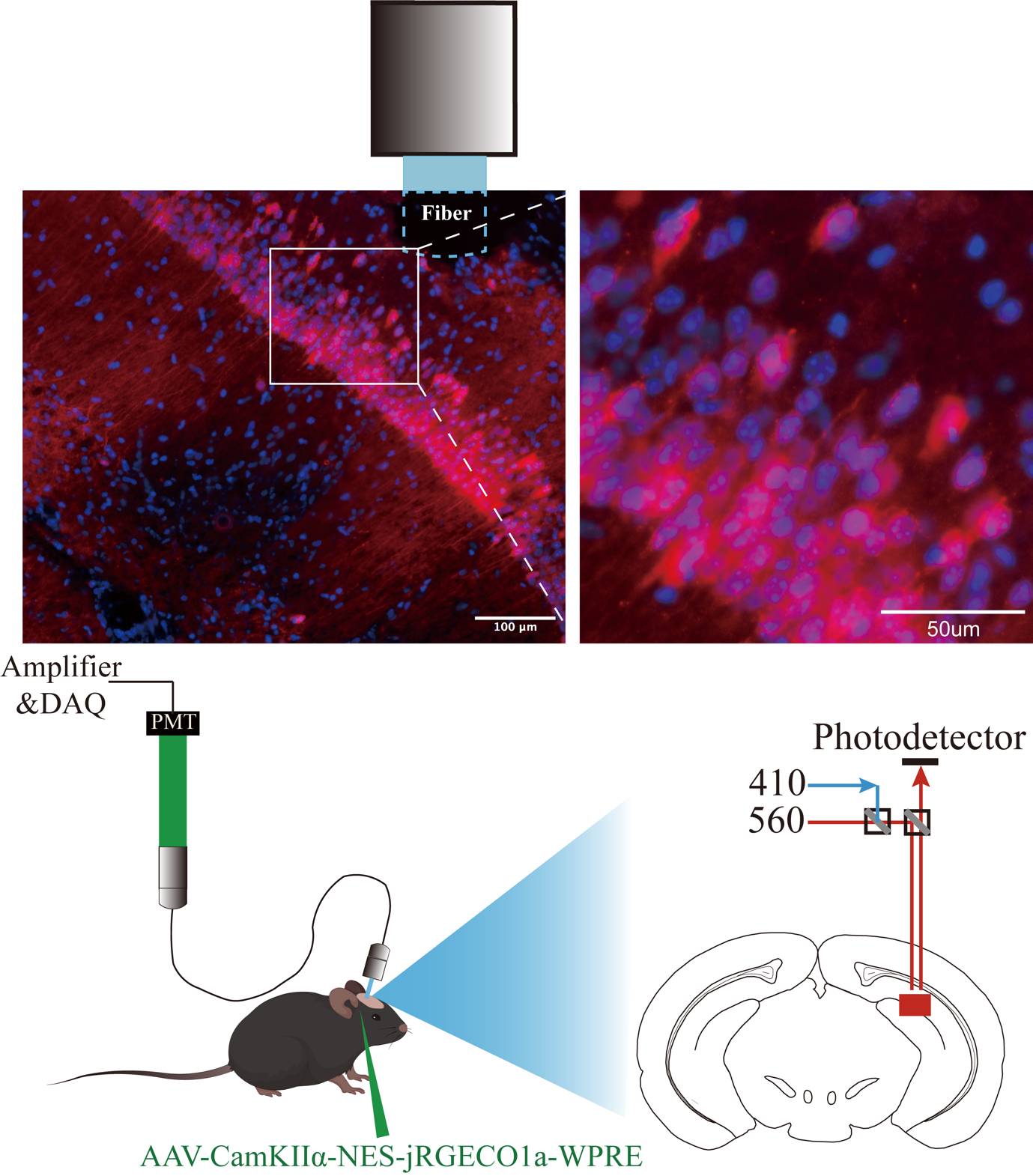


**Figure S7. Localization of the calcium imaging virus (AAV-CamKIIα-NES-jRGECO1a-WPRE) and optic fiber placement.** Red indicates AAV-CamKIIα-NES-jRGECO1a-WPRE expression, and blue indicates DAPI staining. Scale bar = 100 μm (overview images); 50 μm (higher-magnification views).


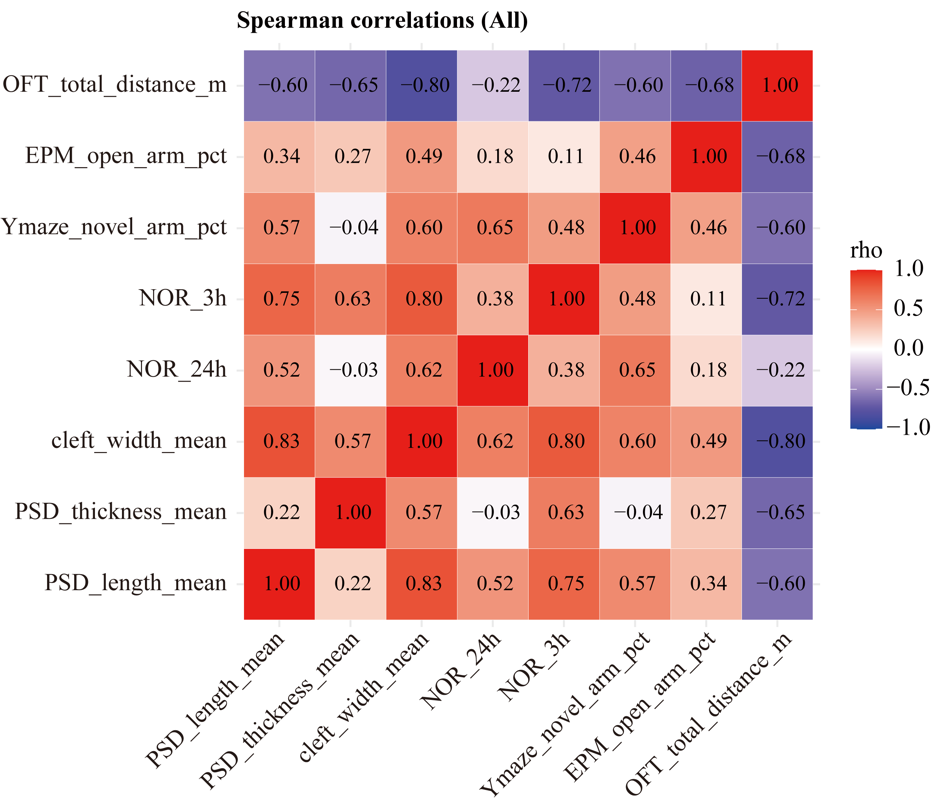


**Figure S8. Exploratory structure-behavior relationships.** Pooled (per-mouse averaged) Spearman correlation matrix across three TEM metrics (PSD_length_mean, PSD_thickness_mean, cleft_width_mean) and five behavioral measures (NOR_3h_RI, NOR_24h_RI, Y-maze novel-arm, EPM open-arm, OFT total distance). Numbers denote Spearman’s rho; asterisks mark FDR-adjusted significance. Complete correlation coefficients and p-values are provided in Supplementary-Statistical results.

**
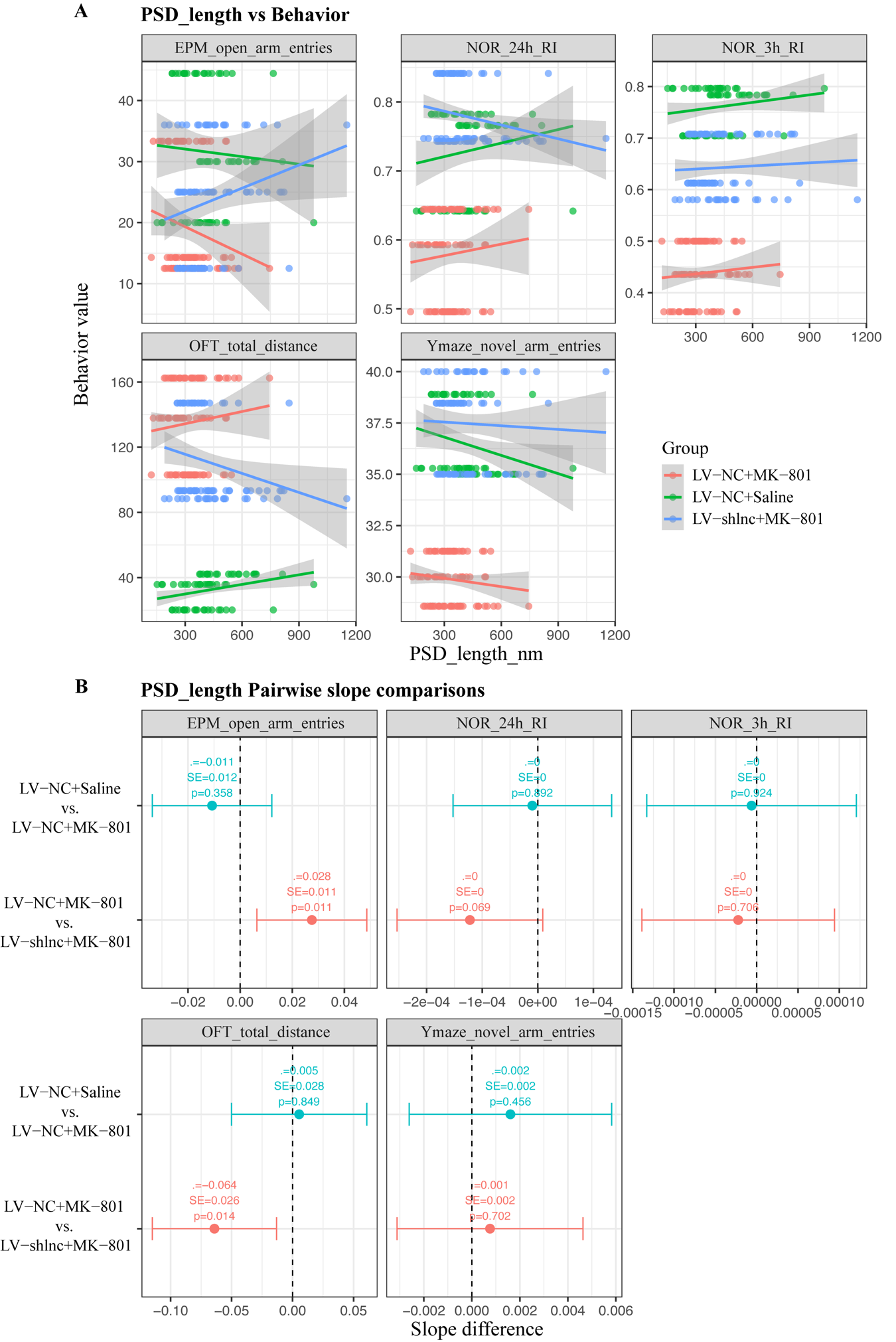
**

**Figure S9. PSD_length-behavior associations across groups.** (A) Scatter plots showing the relationship between PSD_length and behavioral measures (EPM open arm entries, NOR 24h RI, NOR 3h RI, OFT total distance, Y-maze novel arm entries) in LV-NC+Saline (green), LV-NC+MK-801 (red), and LV-shlnc+MK-801 (blue) groups. Lines represent group-specific regression fits with 95% CIs. (B) Forest plots of pairwise slope comparisons between groups (LV-NC+Saline vs. LV-NC+MK-801; LV-NC+MK-801 vs. LV-shlnc+MK-801). Points show slope differences with 95% CIs and p-values, indicating group-dependent alterations in PSD_length-behavior coupling. Significant slope differences suggest that MK-801 alters the relationship between synaptic ultrastructure and behavior, while THUMPD3-AS1 knockdown partially restores this association.

**Supplementary-Detailed Methods**

**Human participants**

Peripheral blood samples from two independent cohorts of Han Chinese individuals were collected, comprising a discovery cohort and a validation cohort. The discovery cohort consisted of 6 healthy human controls and 6 individuals diagnosed with SCZ, whose blood RNA samples were subjected to whole-transcriptome sequencing. The validation cohort comprised 12 individuals diagnosed with SCZ and 12 unrelated non-psychotic controls, whose blood RNA samples were analyzed using RT-qPCR. All participants met the diagnostic criteria for SCZ as outlined in the Diagnostic and Statistical Manual of Mental Disorders (American Psychiatric Association, 4th edition). PANSS scores were available for 18 SCZ patients (Supplementary-PANSS). The non-psychotic controls had no current, past, or family history of psychiatric disorders or substance abuse diagnoses. This study was conducted in compliance with the ethical standards of the ethics committee of Xi’an Jiaotong University. Before the study, all participants were provided with an informed consent form and were briefed on the nature of the procedures before signing.

**Animals**

In this study, male C57BL/6J mice (5 weeks old) were obtained from Chengdu Dashuo Experimental Animal Co., Ltd. Before experimental procedures, all animals were acclimated for 7 days and randomly assigned to experimental groups. The mice were housed under standard laboratory conditions, which included a 12-hour light/dark cycle, a constant temperature of 23 ± 2°C, and a relative humidity of 50 ± 10%. Food and water were provided ad libitum. All animals were monitored daily, and cage bedding was refreshed twice a week to ensure a clean and healthy environment. No procedures were conducted on the day of cage change to avoid confounding stress effects. All animal procedures were conducted in strict accordance with the Guidelines for Reporting of In Vivo Experiments and were approved by the Animal Welfare Committee of Xi’an Jiaotong University.

**Whole transcriptome sequencing analysis**

To investigate the possible ceRNA regulation axis, we conducted whole transcriptome RNA sequencing (RNA-seq) on peripheral blood samples from six SCZ patients (SCZ group) and six healthy controls (HC group). One of the control samples was eliminated due to substandard results during the sequencing process. Total RNA was extracted from the peripheral blood using Trizol reagent (Cat#9109, Takara Bio Inc., Shiga, Japan). Total RNA was analyzed using a NanoDrop 2000 spectrophotometer (Thermo Fisher Scientific, USA) and Agilent 2100 Bioanalyzer (Agilent Technologies, USA) to assess the concentration and quality of RNA. Purified RNA was stored at -80°C.

To comprehensively profile the transcriptome, we performed both mRNA sequencing and small RNA sequencing on the same samples. Briefly, cDNA libraries for mRNA-seq were prepared using the Illumina TruSeq Stranded mRNA Library Prep Kit v2 (Cat#RS-122-2001/2). Small RNA libraries were prepared using the Illumina TruSeq Small RNA Library Preparation Kit (Cat# RS-200-0012). All libraries were sequenced on an Illumina HiSeq 2500 platform, generating 150 bp paired-end reads for mRNA and 50 bp single-end reads for small RNAs. Differentially expressed mRNAs and small RNAs between the SCZ and HC groups were identified using DESeq2 with a threshold of |log2FC| > 2 and p < 0.05.

**Construction of pipeline ceRNAxis**

The pipeline is divided into two major parts: the establishment of a ceRNA reference database and the construction of the lncRNA-miRNA-mRNA network. The interactions between lncRNAs, miRNAs, and mRNAs were extracted from the curated ceRNA reference database. This included known ceRNA relationships derived from experimental data and computational predictions, with each interaction being associated with expression data obtained from the whole transcriptome sequencing analysis. To focus on disease-relevant interactions, a subnetwork was constructed by retaining only the differentially expressed genes from the ceRNA reference network. This process ensured that the interactions selected for further analysis were biologically relevant to the disease context. The filtered ceRNA subnetwork was subsequently analyzed to identify key regulatory axes.

The lncRNA-miRNA-mRNA regulatory axes were screened by applying specific correlation-based criteria: lncRNAs and miRNAs were considered for inclusion in the regulatory axis if they showed a negative correlation. lncRNAs and mRNAs were retained if they demonstrated a positive correlation. miRNAs that interacted exclusively with either mRNAs or lncRNAs, but not both, were excluded from the network, ensuring that only miRNAs with dual interactions in the lncRNA-miRNA-mRNA network were retained. These criteria allowed the identification of relevant lncRNA-miRNA-mRNA regulatory axes that are likely to play a significant role in the disease mechanisms. Moreover, ceRNAxis provides functionality to examine the expression profiles of genes within the selected ceRNA axis between disease and control groups. In addition, by incorporating SNP GWAS data, ceRNAxis can identify SNP mutations affecting components of the selected ceRNA axis.

To evaluate the robustness of ceRNAxis, we systematically compared it with representative ceRNA axis prediction tools. We first conducted a literature and database search (PubMed, Web of Science, GitHub) and identified eight existing methods (DLRAPom ^[1]^, GKLOMLI ^[2]^, ceRNAshiny ^[3]^, ceRNAR ^[4]^, and four additional network-based approaches ^[5-8]^). Two datasets were used for evaluation: our SCZ cohort and a public GDM dataset from the original DLRAPom publication.

**Cell culture and transfection**

The immortalized mouse neuron cell line Cath.a was a gift to Prof. Chunxia Yan. The HEK293 cell line was obtained from the National Collection of Authenticated Cell Cultures (strain GNHu43). Both cell lines were maintained in Dulbecco’s Modified Eagle Medium (DMEM; Cat#L110KJ, Yuanpei, China) at 37°C in a 5% CO₂ atmosphere. Cells were routinely passaged 2-3 times per week using 0.25% trypsin-EDTA (Cat#T1033, New Cell & Molecular Biotech Co., Ltd., China).

To modulate lncRNA THUMPD3-AS1 expression, cells were seeded in 6-well plates and subjected to lentiviral transduction upon reaching 60-70% confluence. The lentiviral vectors (synthesized by Jikai Gene Chemical Technology Co., Ltd., Shanghai, China) included: LV-shTHUMPD3-AS1 (LV-shlnc) for knockdown and a corresponding negative control (NC) lentivirus. Transduction was performed according to the manufacturer’s protocol, supplemented with a lentiviral infection enhancement reagent. The efficiency of overexpression and knockdown was confirmed by measuring lncRNA THUMPD3-AS1 levels 48-72 hours post-transduction.

For rescue and mechanistic experiments, HEK293 or Cath.a cells were co-transfected with miRNA regulators and specifically constructed plasmids (synthesized by Tsingke Biotechnology Co., Ltd., Beijing, China). The transfection mixtures included miRNA modulators and expression plasmids. The miRNA modulators included miR-485-5p mimic, NC mimic, miR-485-5p inhibitor, or NC inhibitor, diluted in DEPC water. Expression plasmids included shTHUMPD3-AS1 (for knockdown), shARHGAP8 (for knockdown), or pcARHGAP8 (for overexpression). When cells reached 60-70% confluence, co-transfection was carried out using an appropriate transfection reagent according to the manufacturer’s instructions. After 4-6 hours of incubation, the medium was replaced with fresh complete culture medium. Cells were harvested 24-48 hours post-transfection for subsequent RNA or protein extraction to analyze the expression of lncRNA THUMPD3-AS1, miR-485-5p, and ARHGAP8.

**Dual-luciferase assays**

The THUMPD3-AS1-WT-3’UTR containing the miR-485-5p predicted target sequence was amplified by PCR from cDNA and subsequently cloned into the pmirGLO dual luciferase vector using Xho I and Sac I sites. A schematic diagram of the pmirGLO vector backbone was generated de novo using Adobe Illustrator (Adobe Inc.), based on the canonical sequence and annotation obtained from SnapGene software (GSL Biotech LLC) and verified against the manufacturer’s documentation. The binding site for miR-485-5p in the THUMPD3-AS1-3’UTR was predicted using TargetScan and RNAHybrid in miRwalk2.0. Mutant THUMPD3-AS1 was generated by introducing mutations in the seed-binding region of the THUMPD3-AS1-WT expression vector. Similarly, we synthesized ARHGAP8-WT-3’ UTR containing the miR-485-5p predicted target and its corresponding mutant. The plasmids were synthesized by Beijing Tsingke Biotechnology Co., Ltd. HEK293 cells were cultured in complete DMEM (containing 10% fetal bovine serum and 1% penicillin-streptomycin). Transfection was performed in Opti-MEM using Lipofectamine 2000 (Cat#11668019, Thermo Fisher Scientific). Following 4-6 hours of exposure to the transfection complexes, the medium was changed back to complete DMEM, and cells were incubated for an additional 48 hours prior to harvest. Dual luciferase activity was measured using the Dual-Luciferase Reporter Assay Kit (Cat#DL101-01, Vazyme, China). The ratio of Renilla luciferase activity to firefly luciferase activity was calculated. All luciferase readings were obtained from more than six separate biological replicates. These experiments were conducted following established protocols for molecular cloning and luciferase assays in molecular biology research.

**Active Rho GTPase Pull-Down Assay**

The activities of RhoA, RhoB, and RhoC were assessed using the Active Rho Pull-Down and Detection Kit (Cat# P2065S, Beyotime, China) according to the manufacturer’s protocol. Briefly, HEK293 cells were lysed on ice using the provided lysis buffer supplemented with protease inhibitors. Lysates were clarified by centrifugation at 14,000 × g for 5 minutes at 4 °C, and equal amounts of protein (600 μg) were incubated with Rhotekin-RBD agarose beads for one hour at 4 °C with gentle agitation to precipitate GTP-bound (active) Rho proteins. After washing the beads three times with wash buffer, bound proteins were eluted by boiling in SDS loading buffer and subjected to SDS-PAGE, followed by Western blotting. Membranes were probed with specific primary antibodies against RhoA (Cat#A0272, 1:1000, ABclonal, China), RhoB (Cat#14326-1-AP, 1:1000, Proteintech, China), and RhoC (Cat#10632-1-AP, 1:1000, Proteintech, China), and detection was performed using HRP-conjugated secondary antibodies (Cat#GB23303, 1:10000, Servicebio, China) and enhanced chemiluminescence (Omni-ECL™ Ultrasensitive Chemiluminescent Detection Kit (Femtocell Grade), Epizyme, China). Input controls (total lysates) were also analyzed to normalize for total protein levels. The assay was conducted across multiple experimental conditions, including control, ARHGAP8 overexpression, and various siRNA knockdown groups (RhoA, RhoB, RhoC, or RhoB/C combined knockdowns), to evaluate differential activation levels of Rho GTPases.

**Protein Domain Annotation and Multiple Sequence Alignment Analysis**

To investigate the structural differences and sequence conservation among RhoA, RhoB, and RhoC proteins, the amino acid sequences of human RhoA (UniProt ID: P61586), RhoB (UniProt ID: P62745), and RhoC (UniProt ID: P08134) were retrieved from the UniProt database. Conserved domain annotation was performed using the NCBI Conserved Domain Database (CDD) to identify key functional regions, including the GTP/Mg²⁺ binding sites, Switch I, Switch II, and the Insert domain. Subsequently, multiple sequence alignment was performed using the ClustalW plugin in MEGA 11, and the resulting alignment was visualized using WebLogo version 3.7.9. In the sequence logo, the horizontal axis represents the amino acid position, while the vertical axis indicates conservation measured in bits. The height of each amino acid letter is proportional to its relative frequency at that position, reflecting the degree of sequence conservation.

**F-actin Cytoskeleton Staining**

F-actin staining was performed in HEK293 cells transfected with ARHGAP8 overexpression plasmid, with or without siRNA-mediated knockdown of RhoA, RhoB, and RhoC. F-actin cytoskeleton organization was visualized using the Actin-Tracker Green-488 probe (Cat# C2201S, Beyotime, China) following the manufacturer’s instructions. HEK293 cells were cultured on glass coverslips in 24-well plates and fixed with 4% paraformaldehyde (Cat#P1110, Solarbio, Beijing, China) for 15 minutes at room temperature. After washing with phosphate-buffered saline (PBS) (Cat#P1020, Solarbio, Beijing, China), cells were permeabilized with 0.1% Triton X-100 (Cat#T9284, Sigma-Aldrich, USA) in PBS for 5 minutes. Following three washes with PBS, cells were incubated with Actin-Tracker Green-488 working solution in the dark for 30 minutes at room temperature to label F-actin filaments specifically. Mounted with antifade mounting medium containing DAPI (Cat#CD110, Zhonghui Hecai, China) for 5 minutes. Coverslips were then mounted and imaged using a fluorescence microscope (Leica, Germany). Stress fiber length and crosslinking density were quantified using ImageJ. Three independent experiments were conducted, with three randomly selected fields per coverslip.

**RT-qPCR, western blotting, and immunofluorescent staining**

In this study, we employed the real-time quantitative PCR (RT-qPCR) technique to detect the expression levels of RNA. The RT-qPCR reaction system consisted of SYBR Green Pro Taq HS Premix qPCR Kit (Cat#AG11701, Accurate Biology, China), primers, cDNA, and deionized water. For cDNA synthesis, we used the Evo M-MLV Reverse Transcriptase Kit (Cat#AG11705, Accurate Biology, China). The miRNA first-strand cDNA was synthesized using the miRNA cDNA First Strand Synthesis Kit (Cat#AG11717, Accurate Biology, China). The Bio-Rad Real-Time PCR System (Bio-Rad, USA) was used to run the RT-qPCR reactions and analyze the data. Relative RNA expression levels between treatment groups were calculated using the comparative 2^-ΔΔCt^ method. GAPDH (for mRNA/lncRNA) or U6 (for miRNA) was used as the internal control.

At the end of the behavioral test, the mice were killed by decapitation, and the brains were quickly removed and placed on ice. The bilateral ventral hippocampus (vHip) was dissected and stored at -80°C. Mice were treated with anti-ARHGAP8 (Cat#PA5-120334, 1:1000, Thermo Fisher Scientific Inc., USA), anti-ROCK2 (Cat#CY6985, 1:2000, Abways, China), anti-ROCK2 (phospho S1366) antibody (Cat#ab228008, 1:1000, Abcam, UK), anti-PSD95 (Cat#ET1602-20, 1:2000, HUABIO, China), Synaptophysin (SYN) (Cat#ET1606-56, 1:5000, HUABIO, China), anti-NMDAR2B (Cat#HA722284, 1:2000, HUABIO, China) and anti-GAPDH (Cat#GB11002-100, 1:5000, Servicebio, China) were used as loading controls. HRP-conjugated rabbit secondary antibody (Cat#GB23303, 1:10000, Servicebio, China). All western blots were quantified using ImageJ Lab (Bio-Rad Laboratories, Hercules, CA, USA).

Ninety minutes after the last behavioral test, mice were perfused intracardially with PBS followed by 4% paraformaldehyde. Brains were post-fixed for 4 hours and dehydrated in 30% sucrose at 4°C for 24 hours. Coronal sections (15 µm) were cut using a cryosectioning machine (Leica, Mannheim, Germany). For c-fos staining, sections were incubated with a c-fos primary antibody (Guinea pig pAb, 1:400, Cat#OB-PGP080-02, OasisBiofarm, China) and detected using a goat anti-Guinea pig IgG(H+L) secondary antibody conjugated to 488 nm fluorophore (Cat#G-GP488, 1:400, OasisBiofarm, China). For ARHGAP8 immunostaining, sections were incubated with the primary antibody anti-ARHGAP8 (Rabbit pAb, 1:50, Cat#NBP1-76568, Novus Biologicals, China) followed by a goat anti-rabbit Cy3-conjugated secondary antibody (Cat#GB21303, 1:400, Servicebio, China). Neuronal nuclei were counterstained with DAPI (Cat#CD110, ZHHC, China). Immunofluorescence images were captured using a fluorescence microscope (Carl Zeiss, Jena, Germany).

**Transmission electron microscopy (TEM) analysis**

First, the tissue was perfused with physiological saline or PBS to remove blood and other impurities. Following this, the brain was perfused with 2.5% glutaraldehyde (Cat#G5882, Sigma-Aldrich, USA) in PBS (Cat#P1020, Solarbio, Beijing, China), maintaining a steady perfusion flow rate. After perfusion, the vHip was immediately dissected and immersed in 4% glutaraldehyde solution (pH 7.4) at 4°C for 4 hours. Once fixation was complete, the tissue was washed with PBS to remove excess fixative. Subsequently, the tissue was washed three times for 10 minutes each with PBS (0.1м, pH 7.4) to remove any residual fixative. The samples were then sent to the Electron Microscopy Facility, Xi’an Jiaotong University School of Medicine, for dehydration, embedding, sectioning, and imaging. Micrographs were analyzed to assess the ultrastructural features of the vHip.

**Golgi staining and image analysis**

Golgi staining was performed according to the method of Zaqout et al ^[9]^. Briefly, brains from 3 independent pairs of different groups were harvested intact and rinsed with distilled water. Brains were incubated in tissue maceration solution for 24 hours in the dark at room temperature. The brains were then transferred to fresh tissue impregnation solution and incubated in the dark at room temperature for 7 days. The brains are then transferred to tissue protection solution and stored at 4°C for 24 h away from light, followed by 7 days of storage in the dark at room temperature. They were cut into coronal sections of 150 μm thickness using a vibratory Slicer mounted on 3% gelatin-coated slides and dried at room temperature and protected from light for 3 days. Dehydration staining was then performed according to the experimental procedure. Stacked images were obtained with a 100× oil immersion lens on a microscope. Image analysis and quantification were performed using Fiji software.

**Stereotaxic surgery**

For the miR-485-5p intervention experiment, brain stereotactic injection of virus into the brain, C57BL/6 mice were anesthetized with sodium pentobarbital (50 mg/kg, intraperitoneal) and placed in a stereotactic device. AAV-U6-miR-485-5p-CMV-EGFP-WPRE-SV40-pA (serotype AAV2/9, 5.4×10^12^ vg/mL, 3*80 nl) was stereotactically delivered into the vHip at the following coordinates (Coordinates: Anteroposterior (AP): -3.5, Mediolateral (ML): ±3.4, Dorsoventral (DV): -4.4/-3.7/-3.1, relative to Bregma). In the THUMPD3-AS1 intervention experiment, LV-shlnc (1×10^8^ TU/mL, 3*130 nl) was delivered stereotactically into vHip at the same coordinates: virus was injected at 0.05 µl/min, followed by a 10-minute rest to ensure optimal virus spread. To knockdown THUMPD3-AS1, two short hairpin RNAs (shRNAs) were designed to target distinct sequences within THUMPD3-AS1. The target sequence for shRNA-#1 was 5’-GGACAGGACAGTGCTTGTTTA-3’ (GC% 47.62%), and for shRNA-#2 was 5’-GCCATTTATGTTCTCAGAAGC-3’ (GC% 42.86%). These shRNA sequences, along with a scrambled negative control sequence (5’-TTCTCCGAACGTGTCACGT-3’, designated CON313), were individually cloned into the lentiviral expression vector GV493 (GeneChem, Shanghai, China). The GV493 vector features a hU6 promoter driving shRNA expression, and a CBh promoter driving the expression of green fluorescent protein (gcGFP) and puromycin resistance gene (IRES-puromycin) for selection. The adeno-associated virus was synthesized by Pivotal Technology Ltd (Wuhan, China), and the lentivirus was synthesized by GKN Bio Ltd (Shanghai, China).

**Fiber photometry calcium imaging**

To monitor neuronal activity in the vHip, we employed a multi-channel fiber photometry system (RWD Life Science, China) to record calcium-dependent fluorescence signals in freely moving male C57BL/6J mice. Mice were anesthetized with 1.5-2% isoflurane and secured in a stereotaxic apparatus. A calcium indicator virus, AAV-CaMKIIα-NES-jRGECO1a-WPRE (serotype AAV9, 9.95×10^12^ vg/mL), produced by OBiO Technology (Shanghai) Corp., Ltd., following standard quality control procedures, was injected into the vHip at a rate of 50 nL/min using a Hamilton syringe, with a total volume of 240 nL delivered. The CaMKIIα promoter ensured selective expression in excitatory neurons. After injection, a ceramic needle was implanted 0.1-0.2 mm above the injection site and fixed with dental cement. Mice were allowed to recover for at least 3 weeks before recording.

In vivo calcium signals were recorded using a multi-channel fiber photometry system (RWD Life Science, China). Excitation light at 410 nm (calcium-independent reference) and 560 nm (calcium-dependent excitation for jRGECO1a) were alternately delivered through the same implanted optical fiber. The emitted red fluorescence was collected via the same fiber, filtered, and detected by a photomultiplier tube (PMT). Light intensity at the fiber tip was maintained below 30 μW to minimize phototoxicity and photobleaching. Signal acquisition and demodulation were performed using the RWD photometry software. The 410 nm signal was used as a reference to correct for motion artifacts and baseline drift, and the fluorescence change (ΔF/F) was calculated by fitting the 410 nm signal to the 560 nm signal.

**Behavioral procedures**

SCZ primarily includes positive symptoms (rapid movements and stereotyped behavior, often tested with open field behavior) and negative symptoms (mainly social withdrawal, emotional blunting, and anxiety). Therefore, this study used the elevated plus maze (EPM) and open field test (OFT) to assess the anxiety condition of the model animals. The novel object recognition test (NOR) and Y-maze were employed to analyze the recognition memory and spatial memory abilities of the model animals, respectively. The three-chamber social test (TCST) was used to investigate whether the model animals exhibited social withdrawal, emotional blunting, and other behavioral changes.

***(i) Assessment of anxiety-like behaviors***

The EPM test is conducted on a square platform consisting of four 33 × 6 cm boards that are connected to a central platform (6 × 6 cm), which has two opposite closed arms and two open arms. The experimental mice are transported to the behavioral laboratory 3 hours before the test to reduce animal stress. The mice are gently placed in the center of the maze facing the open arms, and the experimenter quickly leaves the mice’s field of view to allow them to explore freely for 5 minutes. Before testing each mouse, the EPM is cleaned with 75% ethanol to avoid any potential odor that may affect the test results. The number of entries into the open and closed arms, the duration of activity in the open and closed arms, and the duration of activity in the central area are recorded using ANY-maze software (Stoelting Company, USA). The fewer the number of entries and the shorter the duration of activity in the open arms, the more prominent the anxiety symptoms of the experimental mice.

The OFT is conducted in a square field (45 × 45 × 30 cm) under dim light. The test field is divided into a central area (28 × 28 cm) and a peripheral area. The mice are placed in the center area and allowed to explore freely for 15 minutes. The experimenter remains quiet and out of the mice’s line of sight during the test. The mice’s movement tracks are recorded and analyzed using ANY-maze software, and the time and distance the mice spend in the central area are measured as an anxiety index. The total distance moved by the mice in the maze is used as a locomotor index. The test equipment is cleaned with 75% ethanol after each trial.

***(ii) Assessment of memory***

The NOR test is a three-stage experiment used to assess memory in rodents. During the training stage, the mice are allowed to explore two identical objects for 5 minutes, and the time spent exploring each object is recorded. During the testing stage, two objects are placed in the box, one familiar and one novel, and the mice are allowed to explore them for 5 minutes. The time spent exploring each object is recorded, and a cognitive index is calculated. A cognitive index higher than 0.5 indicates a preference for the novel object, while a cognitive index of around 0.5 indicates memory impairments.

The Y-maze spatial recognition test consists of two experiments separated by a 2-hour interval. During the first experiment (acquisition phase), one arm is closed, and the animal is allowed to explore the other two arms freely for 3 minutes. Two hours later, the second experiment (recall phase) is conducted with all arms open, and the animal is allowed to freely move in all three arms for 3 minutes. The time and distance spent exploring each arm are recorded, and the number of entries into each arm is counted. Memory-impaired animals will show a decrease in the time and distance spent exploring the novel arm. The final parameters to be reported include the number of entries into each arm, the time spent exploring each arm, and the distance traveled in each arm.

***(iii) Assessment of social behaviors***

The TCST is a three-stage experiment used to assess sociability and social novelty preference in rodents. In the first stage, the animal is placed in the center chamber for 10 minutes to habituate. In the second stage, a stranger mouse (Stranger 1) is placed in one of the side chambers in a metal cage, and an empty metal cage is placed in the other side chamber. The animal is allowed to freely explore all three chambers for 10 minutes, while its behavior is recorded. The number of entries into each chamber and the time spent with Stranger 1 are recorded. A decrease in the time spent interacting with Stranger 1 indicates social withdrawal. In the third stage, a new stranger mouse (Stranger 2) is introduced in the opposite chamber, and the animal is allowed to explore for 10 minutes freely. The number of entries into each chamber and the time spent with Stranger 1 and Stranger 2 are recorded. Decreased time spent with Stranger 2 indicates reduced social novelty preference.

**References**

1 Shen, C., Li, H., Li, M. *et al.*, DLRAPom: a hybrid pipeline of Optimized XGBoost-guided integrative multiomics analysis for identifying targetable disease-related lncRNA-miRNA-mRNA regulatory axes. *Brief Bioinform* (2022): **23**, 2, doi:10.1093/bib/bbac046.

2 Wong, L., Wang, L., You, Z. H. *et al.*, GKLOMLI: a link prediction model for inferring miRNA-lncRNA interactions by using Gaussian kernel-based method on network profile and linear optimization algorithm. *BMC Bioinformatics* (2023): **24**, 1188, doi:10.1186/s12859-023-05309-w.

3 Song, Y., Li, J., Mao, Y. *et al.*, ceRNAshiny: An Interactive R/Shiny App for Identification and Analysis of ceRNA Regulation. *Front Mol Biosci* (2022): **9**865408, doi:10.3389/fmolb.2022.865408.

4 Hsiao, Y. W., Wang, L. & Lu, T. P., ceRNAR: An R package for identification and analysis of ceRNA-miRNA triplets. *PLoS Comput Biol* (2022): **18**, 9e1010497, doi:10.1371/journal.pcbi.1010497.

5 Gao, L., Zhao, Y., Ma, X. *et al.*, Integrated analysis of lncRNA-miRNA-mRNA ceRNA network and the potential prognosis indicators in sarcomas. *BMC Med Genomics* (2021): **14**, 167, doi:10.1186/s12920-021-00918-x.

6 Wang, X. J., Gao, J., Yu, Q. *et al.*, Multi-Omics Integration-Based Prioritisation of Competing Endogenous RNA Regulation Networks in Small Cell Lung Cancer: Molecular Characteristics and Drug Candidates. *Front Oncol* (2022): **12**904865, doi:10.3389/fonc.2022.904865.

7 Wang, Y., Zhou, G., Guan, T. *et al.*, A network-based matrix factorization framework for ceRNA co-modules recognition of cancer genomic data. *Brief Bioinform* (2022): **23**, 5, doi:10.1093/bib/bbac154.

8 Yang, X., Yu, D., Gao, F. *et al.*, Integrative Analysis of Morphine-Induced Differential Circular RNAs and ceRNA Networks in the Medial Prefrontal Cortex. *Mol Neurobiol* (2024): **61**, 74602-4618, doi:10.1007/s12035-023-03859-x.

9 Zaqout, S. & Kaindl, A. M., Golgi-Cox Staining Step by Step. *Front Neuroanat* (2016): **10**38, doi:10.3389/fnana.2016.00038.
